# Translating large-scale climate variability into crop production forecast in Europe

**DOI:** 10.1038/s41598-018-38091-4

**Published:** 2019-02-04

**Authors:** Gabriela Guimarães Nobre, Johannes E. Hunink, Bettina Baruth, Jeroen C. J. H. Aerts, Philip J. Ward

**Affiliations:** 1 Institute for Environmental Studies (IVM), Vrije Universiteit Amsterdam, De Boelelaan 1087, 1081 HV Amsterdam, The Netherlands; 2 FutureWater, Cartagena, Spain; 3 Directorate Sustainable Resources, European Commission, Joint Research Centre, Ispra, Italy

## Abstract

Studies show that climate variability drives interannual changes in meteorological variables in Europe, which directly or indirectly impacts crop production. However, there is no climate-based decision model that uses indices of atmospheric oscillation to predict agricultural production risks in Europe on multiple time-scales during the growing season. We used Fast-and-Frugal trees to predict sugar beet production, applying five large-scale indices of atmospheric oscillation: El Niño Southern Oscillation, North Atlantic Oscillation, Scandinavian Pattern, East Atlantic Pattern, and East Atlantic/West Russian pattern. We found that Fast-and-Frugal trees predicted high/low sugar beet production events in 77% of the investigated regions, corresponding to 81% of total European sugar beet production. For nearly half of these regions, high/low production could be predicted six or five months before the start of the sugar beet harvesting season, which represents approximately 44% of the mean annual sugar beet produced in all investigated areas. Providing early warning of crop production shortages/excess allows decision makers to prepare in advance. Therefore, the use of the indices of climate variability to forecast crop production is a promising tool to strengthen European agricultural climate resilience.

## Introduction

By 2050, the global demand for agricultural goods is expected to grow sharply, driven by the projected demands from an expanding world population, dietary shifts, and increasing biofuel consumption^1–3^. At the same time, there are several major obstacles to boosting crop yields, including a decrease in the area of arable land per person^4^, and variability in global climate. Creating a resilient agricultural system requires the incorporation of preparedness measures against weather-related events that can trigger disruptive risks such as droughts.

The use of climate information with long-lead times, such as the seasonal predictions of the El Niño Southern Oscillation (ENSO), has allowed farmers to anticipate risks and to improve their management in several parts of the world^5–10^. ENSO influences global agriculture in several ways, including through changes in hydro-meteorological extremes^11–16^ and climate extremes^17–20^, which directly or indirectly impact crop yield, production and prices^7,21–23^. However, ENSO only slightly modulates the European climate^24,25^, where the interannual anomalies in common atmospheric variables such as temperature and precipitation are driven mostly by other atmospheric oscillations^11^. For instance, the North Atlantic Oscillation (NAO), the East Atlantic/West Russian pattern (EA/WR) and the East Atlantic Pattern (EA) are known to be related with precipitation patterns in Europe, especially in the Iberian Peninsula^11,26–31^, and the Scandinavian Pattern (SCA) influences rainfall in north-eastern Europe^32^.

Whilst several studies have found connections between these indices of climate variability and common atmospheric variables, few have addressed the role of large-scale atmospheric oscillations on the variability of agricultural production, especially at the pan-European level. Initial studies^33,34^ investigating the relationship between modes of climate variability and winter wheat anomalies concluded that NAO and EA patterns are strong indicators of yearly wheat deviations, and other studies found significant connection between major European crops and indices of large-scale climate variability^35–39^.

Agricultural producers make decisions regularly throughout the year, including tactical ones (actions to be taken within weeks or months) and strategic ones (actions to be taken within future seasons or years)^6,40^. Introducing climate forecasting into producer management depends on the availability of relevant information during the decision-making process, which requires an understanding of the relationship between European climate variability and crop production at several lead times (LD)^6,41–43^. To the best of our knowledge, there is no climate-based decision model that uses indices of atmospheric oscillation to predict agricultural production risks in Europe at different lead times.

In this paper, we develop such a model for multiple time-scales by exploring the relationship between large-scale indices of climate variability and anomalies in sugar beet production. Therefore, we aim at identifying those regions where a robust model can be established based on the indices of atmospheric oscillation investigated. For this, we applied a supervised Machine Learning decision tree-based algorithm^44^, using predictors (in this case the ENSO, NAO, SCA, EA and EA/WR) recorded within the growing season to establish a prediction between high and low values of the predictands (sugar beet production). Based on the accuracy and predictive skill of the model, we also discuss how this information potentially improves the management of the agricultural sector by combining the findings with a seasonal forecasting system of crop production.

## Results

### General performance of the FFT models

In this section, we analyse the performance of the Fast-and-Frugal Trees (FFT) in predicting high/low sugar beet production events. The best performing indices of climate variability for each NUTS2 (Nomenclature of territorial units for statistics) region are shown in the supplementary material (Table S1.1–S1.6 and Fig. S2).

**Figure 1:**
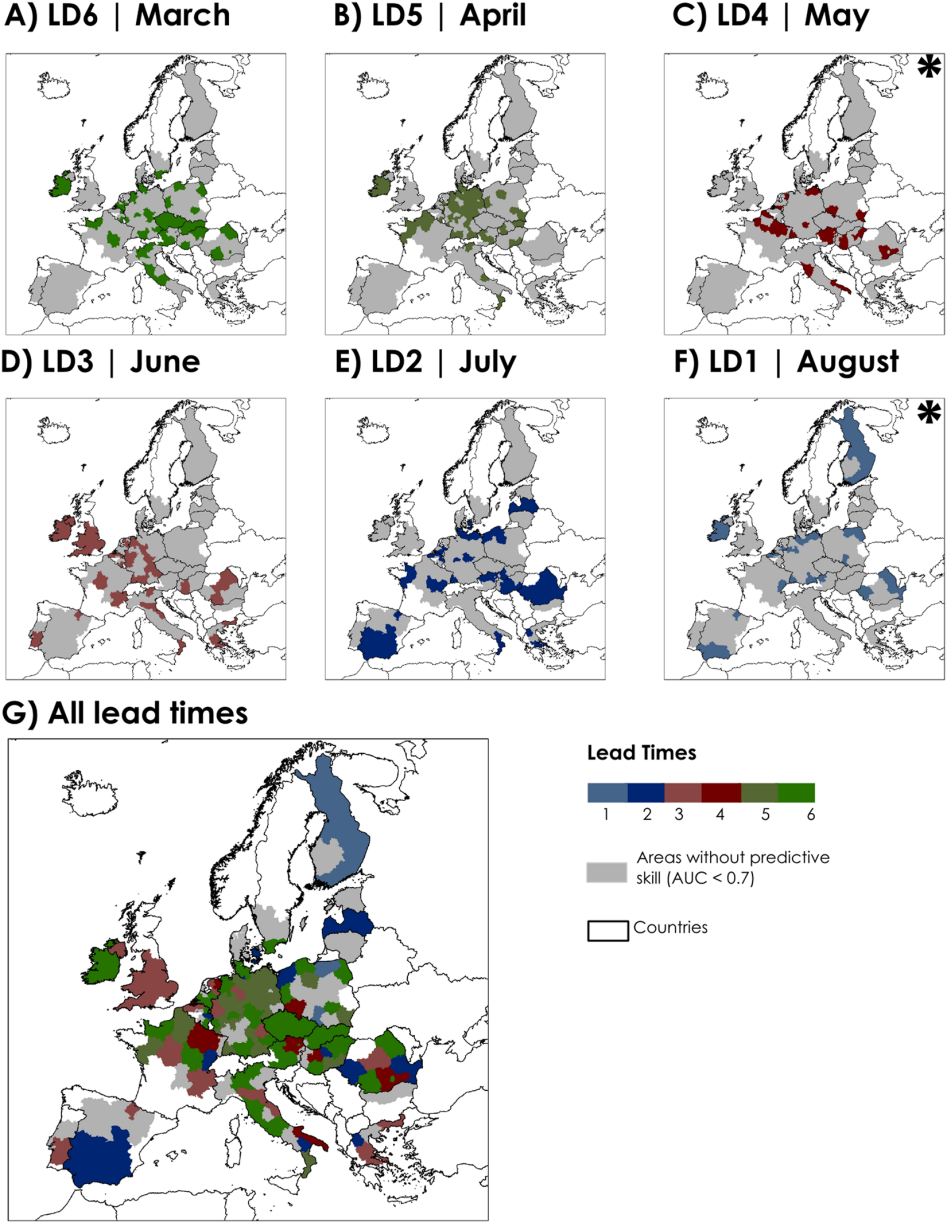
Regions where the FFT models have predictive skill (Area Under the Curve index or AUC > 0.7): (**A**) six months (March) to (**F**) one month (August) before the beginning of the harvesting season. In (**G**) the maps were overlaid in descending order from longest to shortest lead time. Regions without predictive skill (AUC < 0.7) are shown in grey. Field significance of the results was assessed using the binomial distribution and found to be highly significant (P < 0.001). Lead times that were found to be significant only due to bootstrapping (P < 0.1) are indicated with an asterisk.

**Figure 2:**
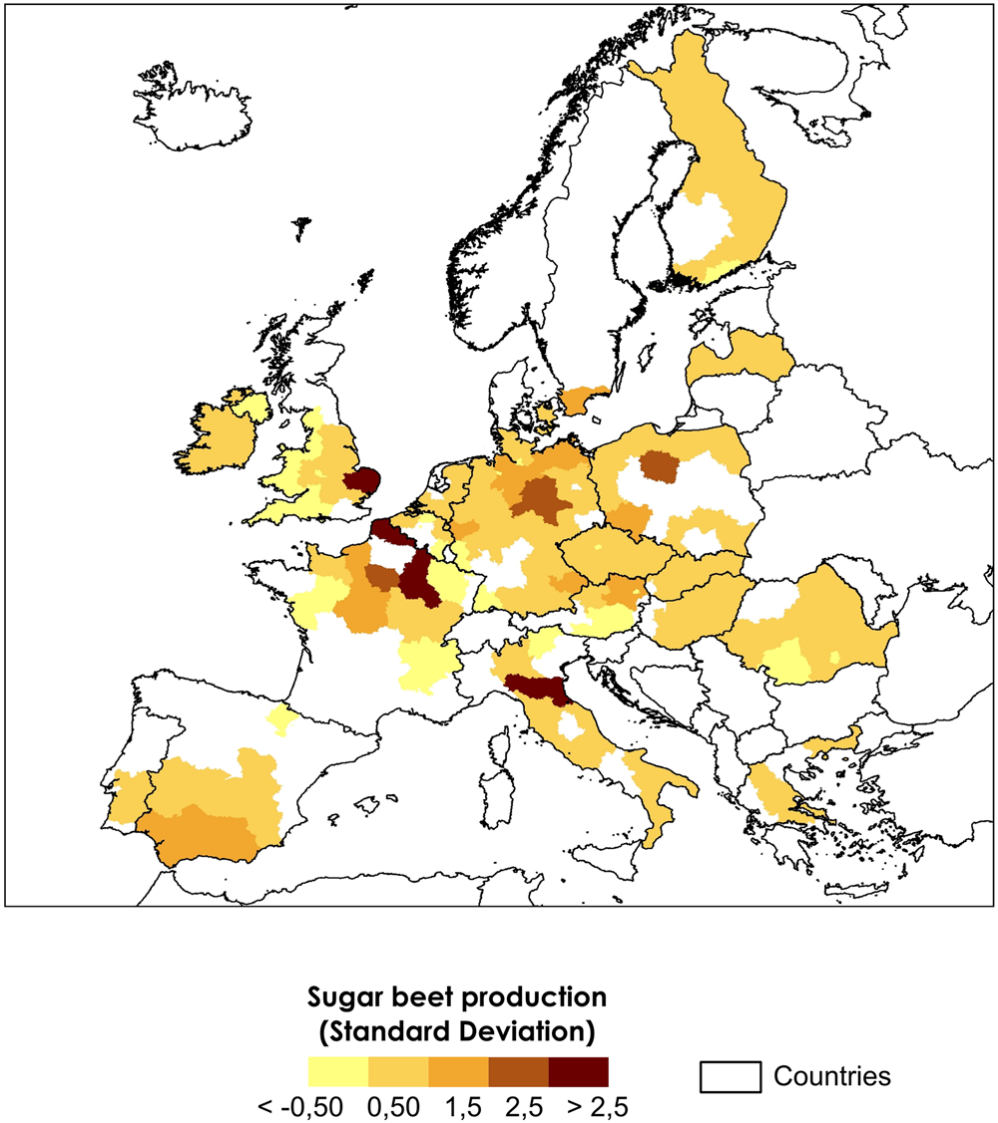
Standard deviation of the mean sugar beet production in NUTS2 regions where the FFT models have predictive skill (AUC > 0.7) in all lead times.

In total, the cross-validated FFT models distinguished between sugar beet high/low production events in 160 out of 207 NUTS2 regions, covering nearly 77% of investigated areas (Fig. 1G); 81% of the mean annual sugar beet production is harvested in these regions (Fig. 2). An overview of the mean sugar beet production in all NUTS2 regions investigated is available in Fig. S3. In some locations, the FFT models have skill to predict high/low production for more than one LD, and already at LD6 (March) before the start of the sugar beet harvesting season.

#### Predicting sugar beet high/low production events

**Figure 3:**
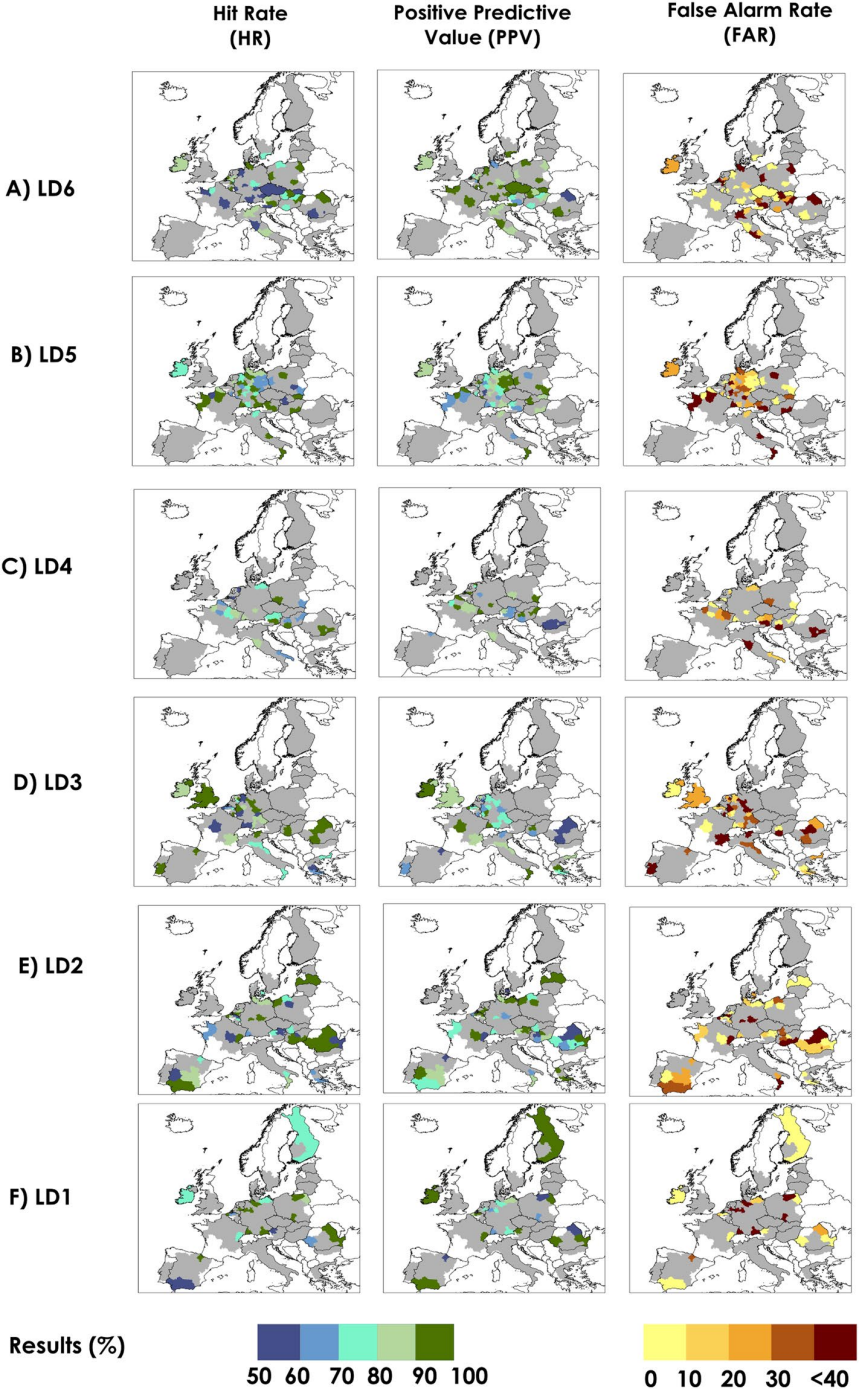
Performance metrics for predicting low production sugar beet events for areas with AUC > 0.7 at six lead times (**A** to **F**). Hit Rate (HR) is the probability of low production occurrences that were correctly predicted; Positive Predictive Value (PPV) index represents the probability of FFT to detect true low production sugar beet events over all low production (including False Alarms); False Alarm Rate (FAR) is the probability of a false low production occurrence. Regions without predictive skill (AUC < 0.7) are shown in grey. The FAR is cut off at <40% because in more than 90% of the NUTS2 regions the results are below this threshold.

**Figure 4:**
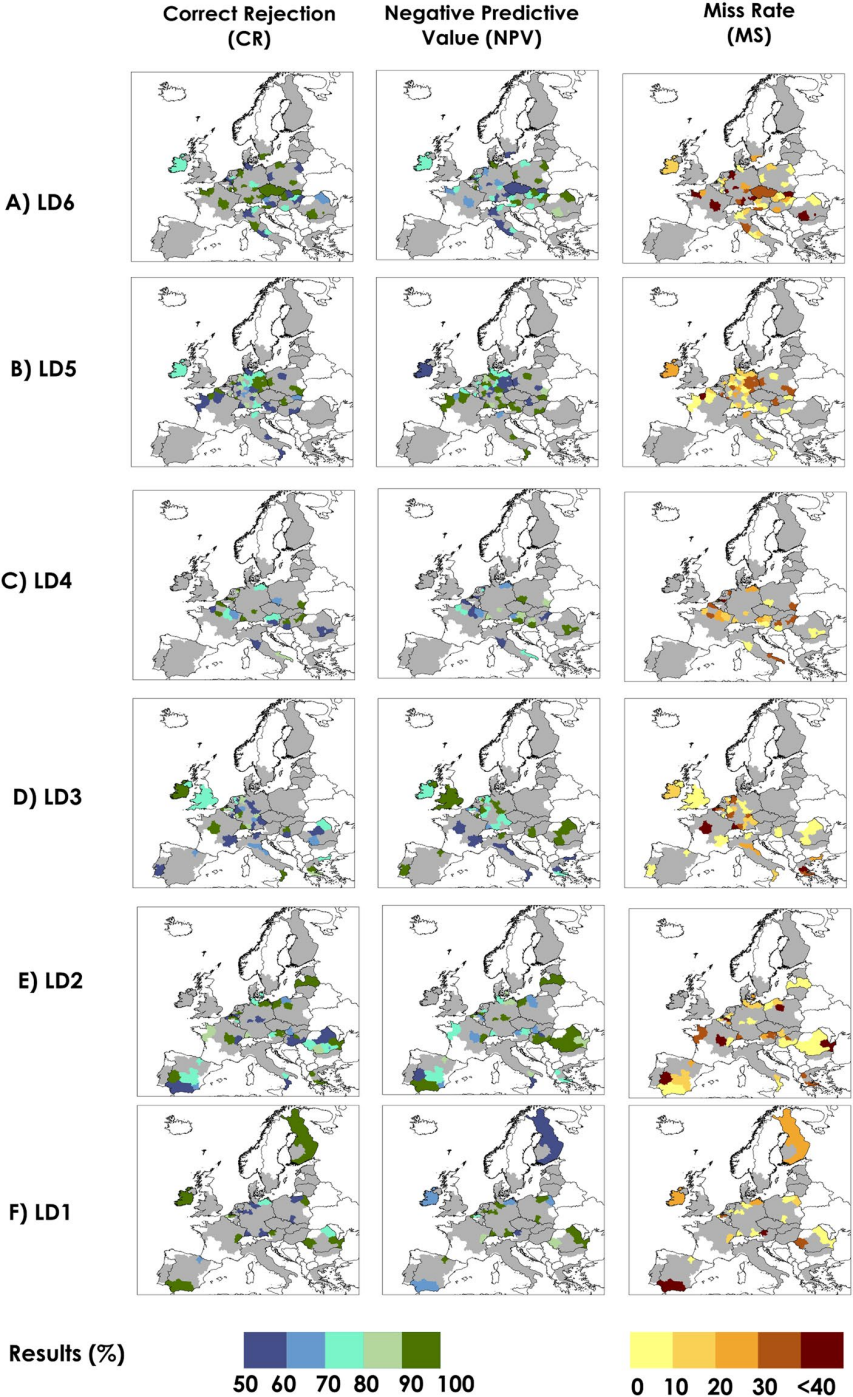
Display of standard classification statistics for predicting high production sugar beet events for areas with AUC > 0.7 at six lead times (**A** to **F**). Correct Rejection Rate (CR) shows the probability of high production occurrences that were correctly predicted; Negative Predictive Value (NPV) index represents the probability of the FFT detecting a true high production sugar beet event over all high production (including Misses); Miss Rate (MS) is the probability of a false high production occurrence. Regions without predictive skill (AUC < 0.7) are shown in grey. The MS is a cut off at <40% because in more than 90% of the NUTS2 regions the results are below this threshold.

In March and April, six (LD6) and five (LD5) months before sugar beet harvesting in Europe respectively, we found that the FFT models have predictive skill in a total of 79 out of 207 NUTS2 regions, with an Area Under the Curve (AUC) index ranging from 0.70 to 1.00 (Supplementary Table S1.1 and S1.2). Western and eastern Europe have the highest number of NUTS2 regions with AUC > 0.7. In 56% and 55% of the NUTS2 regions located in western and eastern Europe respectively, significant predictions (P < 0. 1) are already observed in these lead times. For LD6 and LD5, the overall balanced accuracy (BACC), which represents the skill of the FFT in correctly predicting high/low production events, is on average, 79% for both lead times. Approximately 44% of the mean annual sugar beet is produced in these 79 NUTS2 regions. The Hit Rate (HR), thus the probability of a predicting a true low sugar beet production event in these regions is, on average, 74% and 84% at LD6 and LD5, respectively. The spatial distribution of the HR, False Alarm Rate (FAR) and Positive Predictive Value (PPV) are shown in Fig. 3. The HR values are especially high (above 90%) in central Europe, particularly over large areas in Germany. On average, the probability of predicting a false low sugar beet production event (FAR) is 17% and 26% at LD6 and LD5, respectively. The probability of a true low production (hereafter referred to as PPV) over all low production events predictions represents the trade-off between HR and FAR. PPV values are, on average, 90% and 83% at LD6 and LD5, respectively. In Fig. 4, the results are presented for predicting high sugar beet production events. We found that the probability of predicting a true high sugar beet production event in these regions (defined as Correct Rejection Rate or CR) is, on average, 83% and 74% at LD6 and LD5 respectively (Fig. 4A,B). The probability of predicting a false high sugar beet production event (defined as Miss Rate or MS) is, on average, 26% and 16% at LD6 and LD5, respectively. The average Negative Predictive Value (NPV), which represents the probability of a true high production (CR) over all high production events predictions, is 74% and 81% at LD6 and LD5 respectively. In the supplementary material Fig. S2, we display the respective large-scale indices of climate variability that were used by the FFT models as a predictor of high/low production events; results are shown for areas with significant predictive skill (AUC > 0.7 and P < 0. 1). In March and April, during the early growing stages of sowing and emergence, sugar beet is sensitive to water deficits and frost, and winter and early spring weather conditions in Europe are strongly associated with EAWR, NAO and EA^11,25,35,45,46^. Relationships between sugar beet production and mean EAWR, EA and NAO from January to March, and mean EA and NAO from February to April were found in this study, and also by others when assessing the relationship between these atmospheric oscillations and other crop types, productivity and vegetation dynamics^35,47–49^. In addition, we observed that in most of the regions, multiple indices of atmospheric oscillations were used simultaneously as a predictor of sugar beet production instead of a single index (Figs S2, S4). We observed that winter and early spring NAO influences summer crop production in large areas in Europe, especially in Germany (LD5), as previously found by others^35,50^, where positive NAO in January and February drives more intense precipitation in northern and north western Europe and the opposite in the south of the continent^11,25^, which might affect the early growing season. However, we neither find a north-south dipole impact of the NAO at these lead times, as often is observed for rainfall, nor a large influence of NAO in southern Europe, as found by others^48,51^. Other important predictors of sugar beet high/low production events are EA and EA/WR patterns, especially in regions in western and eastern Europe. Since precipitation and temperature variability in Europe are more strongly modulated by winter and spring oscillation regimes, the relevance of winter and spring atmospheric oscillations may be twofold: (a) winters and early spring modes of climate variability provide soil moisture for crop development in summer, as also suggested by others^50,52,53^; (b) winter and spring weather conditions affect sowing and early growing stage, as found by previous research^35,54^.

In May and June, four and three months before the start of the sugar beet harvesting season, the FFT models have predictive skill in a total of 92 out of 207 NUTS2 regions, as displayed in Fig. 1C, D. Within the investigated areas, approximately 47% of the mean annual sugar beet is produced in these 92 NUTS2 regions. Comparing all lead times investigated, LD3 was found to be the one with the most regions with predictive skill (74 out of 207). In May and June, western and northern Europe have the highest number of NUTS2 regions with AUC > 0.7. In 54% and 67% of the NUTS2 located in western and northern Europe respectively, significant predictions (P < 0. 1) are found in these lead times. In addition to LD6 and LD5, the FFT models extend the predictive skill to some western and southern European regions and the United Kingdom. The AUC index ranges from 0.70 to 1.00 (Supplementary Table S1.3 and S1.4). On average, the mean BACC is 78% (LD4) and 83% (LD3). In these areas, HR, PPV and FAR are, on average, 78%, 86% and 22% (LD4) and 89%, 82% and 27% (LD3) respectively. The CR and NPV are, on average, 78% and 74% (LD4), and 72% and 88% (LD3) respectively. The mean MS at LD4 and LD3 is 22% and 11% respectively (Fig. 4C,D). In June, sugar beet root has reached its vegetative growth, and beet leaves are subjected to both heat and water stress^55^. FFT models primarily used EA/WR and ENSO averages from spring and late spring (Fig. S2 LD3) as the main predictors of sugar beet production in western and eastern Europe. These indices have also been found to have links with maize grain and wheat yield in other studies^7,35^. Spring/late spring weather in Europe are associated with a range of atmospheric oscillations including EA/WR and EA^56^, and less influenced by NAO^11^. The EA/WR pattern influences mainly western and eastern Europe, where a positive (negative) EA/WR phase is related to high temperature anomalies^35^ and drier (wetter) conditions. ENSO was previously found to have significant associations with changes in precipitation in spring, especially over western and northern Europe^11,57^. However, in Spain, where we would expect a larger spatial link between SCA, EA and ENSO patterns on precipitation^11^ and consequently on crop production^35^, the effect of these climate patterns may be weaker than elsewhere, probably due to common irrigation practices in some areas, especially in southern and north western Spain^58–60^.

In July and August, the FFT models predict sugar beet high/low production events in 62 of the 207 NUTS2 regions (Fig. 1E,F), with an AUC index ranging from 0.70 to 1.00 (Supplementary Table S1.5 and S1.6). In July and August, western and eastern Europe have the highest number of NUTS2 regions with AUC > 0.7. In 35% and 41% of the NUTS2 located in western and eastern Europe respectively, significant predictions (P < 0. 1) are found at LD2 and LD1. In addition to models at LD6 to LD3, the FFT models extend the predictive skill to several regions in Europe, especially in large areas of Spain and Finland. On average, the mean BACC for LD2 and LD1 is 80%. Within the investigated areas, approximately 22% of the mean annual sugar beet is produced in these 62 NUTS2 regions. The average HR, FAR and PPV is 81%, 22% and 82% for LD2, and 83%, 22% and 84% for LD1 (Fig. 3E,F). We found that the CR, MS and NPV is, on average, 78%, 19% and 83% for LD2, and 78%, 16% and 81% for LD1. (Fig. 4E,F). In July and August, when the sugar beet has reached its late development stage, reduced water availability has a small impact on the yield. EA/WR from May to July in addition to EA and NAO averages in June to August are most used as sugar beet production predictors in large areas of Europe. In summer, the EA/WR pattern was found in previous research^35^ to have strong links with positive temperature anomalies and below-average rainfall in western and south eastern Europe, which is beneficial for harvesting. However, our results also suggest that multiple indices of atmospheric oscillation show links with sugar beet production in scattered areas in Europe, especially at LD2 (Fig. S2). In addition, previous investigations found that the precipitation, temperature, vegetation dynamics and maize yield in Europe have links with these atmospheric oscillations in summer^11,35,47^. Large-scale atmospheric oscillation indices may have less predictive skill for production in early summer than winter and spring for two reasons: (a) due to the higher importance of regional-to-local atmospheric phenomena during summer, also highlighted by others^35^. This means that sugar beet production estimates might be more efficiently captured by the regional crop model forced by observed surface climate variables locally; and (b) in July and August, when the sugar beet has reached its late development stage, reduced water availability has a small impact on the crop^61^.

## Discussion and Conclusion

In this section we discuss how our findings could be used to support agricultural management and decision-making processes in Europe, followed by recommendations, limitations and conclusions of the study.

Currently, the Joint Research Centre (JRC) MARS crop yield forecasting system (M-CYFS) forecasts national sugar beet yields for the current growing season based on a scenario technique and trend analysis^62^, which substantially differs from the method applied in this study, which also targets production. The M-CYFS is a decision support system with the purpose of providing evidence-based information on the status of annual crops in the EU and neighbouring countries by monitoring crop growth and forecasting crop yields along the season^5,63,64^. It uses agro-meteorological indicators derived from observed meteorological data as well as crop growth models and remote sensing information, which are applied together to build a statistical yield forecast using best-fit criterion to explain a cause-effect relationship with historical yield statistics at national level. Sugar beet forecasts early in the season are purely based on observed trends, but could potentially gain predictive skill if indices of climate variability, such the ones investigated in this study, are integrated in M-CYFS based on the outcomes of this study. In general, agro-meteorological and remote sensing indicators start to demonstrate a certain reliability for the regression forecasts (r-squared value > 0.5 if de-trended data are used) from the end of June or beginning of July depending on the country. Furthermore, since large-scale indices of climate variability can be predicted with higher lead times than weather variables and related crop growth variables, the M-CYFS could further extend its lead-time if predictions of the large-scale indices of climate variability were used from dynamic climate models^35^.

Anomalies in temperature and precipitation driven by climate variability do not always explain crop production, and regions without predictive skill, may have been masked by a number of local agro-management activities. For instance, irrigation practices, which are observed in Spain and Bulgaria, may have lowered the negative impacts of unfavourable weather conditions and the skill of the FFT models in detecting high and low production. Overall, the decision-model performed more consistently in eastern and western Europe. These regions also have low shares of irrigated areas, which may have influenced the model performance^58^. Additionally, in some regions, limitations in data availability on crop production were encountered during this investigation (described in the methods section), and this may have affected the extraction of the high/low sugar beet production indicator per NUTS2 region. For instance, we disaggregated national crop production statistics data in several NUTS2 regions (available in supplementary S7), and our downscaling method adds uncertainty to the model predictions. Therefore, the FFT models may not be performing satisfactorily in some regions for three reasons: (a) presence of irrigation practices, which may inhibit the impacts of unfavourable weather conditions; (b) lack of underlying physical relationship between sugar beet production and the investigated indices of climate variability; and (c) due to limitations in the sugar beet production downscaling method.

In Europe, the growing period of sugar beet is normally between 140 and 160 days^61^, mostly in the northern half of Europe, where the climate is more suited to growing beet^65^. Sugar beet is planted in early spring and harvested in late September, before the cold season starts. In March, beet producers make strategic decisions regarding the amount of sowing for the year and planning tactical actions for the germination and early plant development stages, which comprise the most sensitive periods of the crop^61^. Due to strict regulations of the EU sugar market, the 2007 Sugar Reform limited total EU production to 14.7 million tonnes of raw sugar until the marketing year 2016/2017^65^. If climatic conditions indicate a probable “high production” scenario at this early stage, the EU could better plan “out-of-quota” measures, such as: exporting the excess of sugar beet production to the EU’s annual World Trade Organisation quota, which is limited to 1.374 million tonnes; disposing excess on the EU market for industrial purposes; or counting against the following year’s sugar “quota”^65^. In addition, each year the EU market must decide by March 16^th^ for a first “preventive” withdrawal to allow producers to reduce their beet sowings. However, the quota management ended as of 30 September 2017. On the other hand, if climatic conditions show signs of shortage in production, tactical measures can be taken to increase supplies as follows: (1) better preparation or further investment in responsive irrigation schemes as sugar beet is particularly sensitive to water deficits in early spring^55,66^; (2) taking measures to prevent freeze damage to crops such as active methods (e.g. adding heat and covering crops) and passive methods (e.g. proper scheduling of planting within the safe freeze-free period) as night frost in spring can damage sugar beet and delay seed germination^67,68^; and (3) before planting, producers could decide to reduce their financial losses by purchasing appropriate crop insurance products against deviations from their long-term yields. Prior information about the spatial configuration of risk would support insurance companies to better allocate resources to comply with the EU solvency requirements, which demands that insurers have adequate reserves for 99.5% of potential loss events^69^.

By June, sugar beet root has reached its vegetative growth, and its leaves are subjected to both heat and water stress^55^. Temperatures greater than 30 °C greatly decrease sugar yields^61^, and water stress is considered the major limitation to crop productivity and yield stability^66^. In central and western Europe, drought stress can reduce sugar beet yields by 10–30% compared to the long-term average^70,71^. Beet water requirements are moderate, and if low production is predicted, adequate water should be available to allow the sugar beet to develop a good root system for extracting water from the soil. Predictions of low or high production, mainly needed on a regional scale for industries and policy makers^72^, supports the sugar industry in adapting normative plans for optimizing processing campaigns^73^, such as factory operations concerning delivery schedules and storage capacity, transport logistics and export sales. Sugar beet production forecasts could also be a useful aid for marketing operations, where prices fluctuate based on the supply and demand of the product^72^.

In August, when the sugar beet has reached its late development stage, reduced water availability has a small impact on the yield^61^. Forecasts of low production could support sugar beet producers to better prepare against cold and wet days, which often lead to deterioration of harvesting conditions and increase the probability of fungal infections, increasing the risk of late harvesting in autumn^74^. Moreover, forecasts of high production may indicate favourable conditions to the harvesting period, when certain levels of soil moisture and rain-free days are preferred^74^.

Our study did not aim to produce quantitative prognostic information about crop production; instead, we focused on identifying those regions where a robust model can be established based on the indices of atmospheric oscillation investigated, and used as an early warning indicator for crop impacts. This is a primary step towards the adoption and use of climate-related forecasts in agricultural decision-making: if there were no climate variability influence on crop production, it is unlikely that agricultural stakeholders and markets would benefit from long-lead time climate information. In addition, the observed indices of climate variability assessed in this study can be forecast with varying levels of skill and lead times. Skilful predictions of NAO have been extended to more than a year ahead^35,75^. EA summer and autumn anomalies can be properly hindcast with a lead time of 1 to 2 months^25,76^, while ENSO forecasting is more developed, and most prediction systems have some skill for predicting events up to 14 months lead times^77^. Given that the seasonal predictability of large-scale climate variability is generally higher than that of surface weather variables in Europe^35^, empirical seasonal risk outlooks could potentially be developed based on predicted values of the indices of climate variability^78^.

The current study is a statistical analysis of the effect of climate variability on sugar beet production, and resulted in the selection of related predictors for each region in Europe out of a total of five indices of climate variability. Future work could benefit from using different methods to classify the different “low/high production”, and examining time-lags between the indices of climate variability on the agricultural impact indicators. Moreover, some of the significant results may had occurred by random chance (LD4 and LD1 Fig. 1), and results may be interpreted with caution. Further insights into relationship between climate variability and crop production can be obtained by applying compensatory models since a more complex model can reveal important features that are not being captured by a simple model. In addition, this study can benefit from testing different decision algorithms, since the ifan algorithm, which was adopted in this study, assumes independence between predictors. Furthermore, crop production databases, such as the one used in this study, are also known to face limitations, such as reporting errors. Testing the proposed method on other crop production databases and crop types could provide further insight in the strengths and limitations of the approach. Lastly, climate can only partly explain sugar beet production. Other important aspects such as changes in farm-level management, economy, agronomy and quality of the land were not included in this study.

## Methods

**Figure 5:**
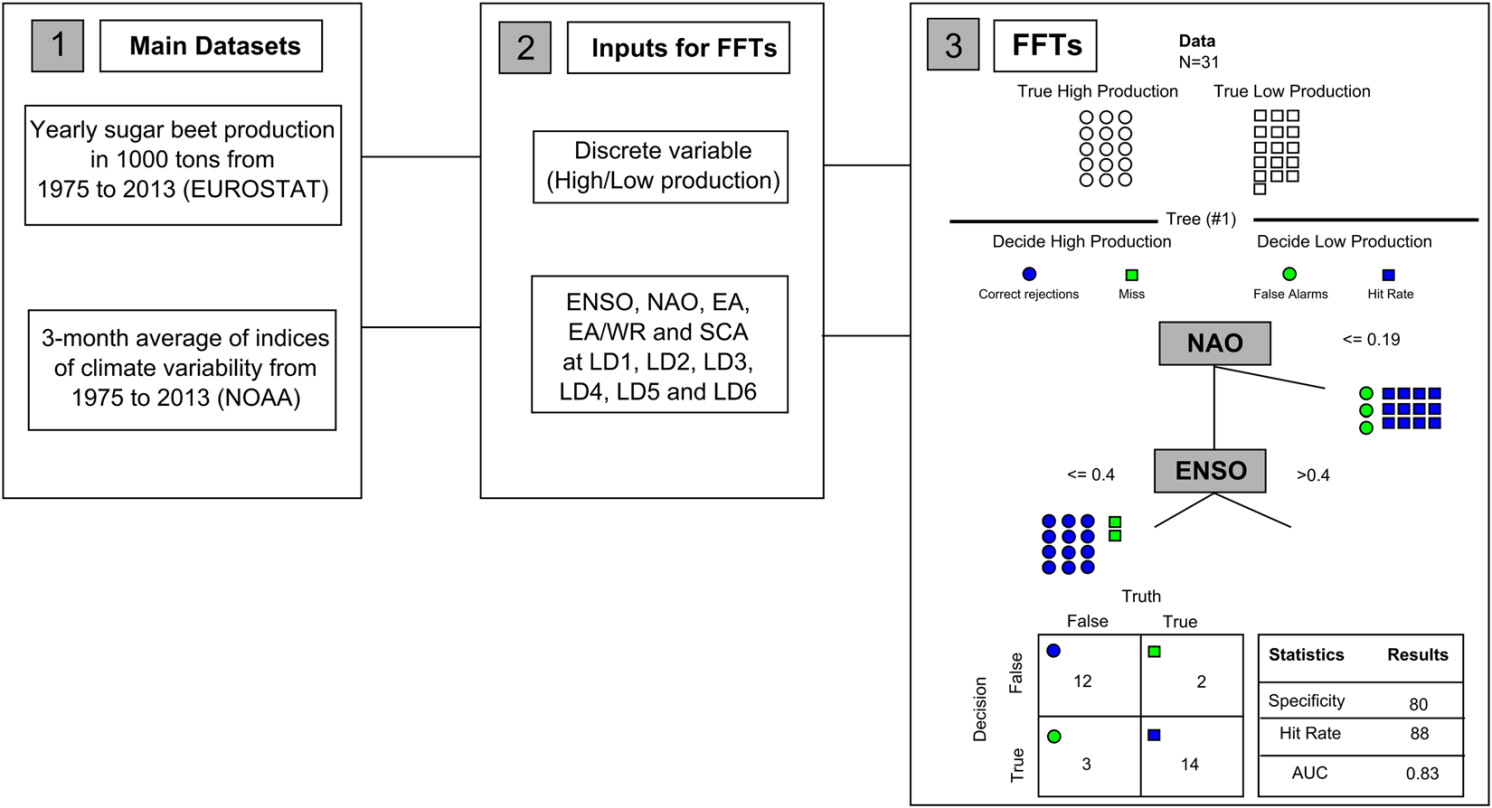
Flowchart representing the methodological framework applied in this study, handled in three steps: (1) collection of two main datasets; (2) extraction of five climate indicators at six lead times, and a discrete variable based on sugar beet climatological anomalies; and (3) example of an FFT output model containing standard classification statistics for a specific NUTS2 region and LD3.

We use FFT^79^ to predict impacts on agricultural production applying five large-scale indices of climate variability: ENSO, NAO, SCA, EA and EA/WR. The FFT models identify which indices of climate variability are capable of classifying production in given years into high or low production classes. From a database of historical sugar beet production of the European statistical office (EUROSTAT), we derived a yearly agricultural production indicator from 1975–2013, namely high/low production based on observed anomalies. For the same period, we obtained 3-month average values for the indices of climate variability from January to March, February to April, March to May, April to June, May to July and June to August, further referred to as LD6, LD5, LD4, LD3, LD2 and LD1, respectively. An overview of the methodological framework is displayed in Fig. 5. The methods and datasets are described in detail in the following subsections.

### Indices of climate variability

In this study, we represent climate variability using a 3-month average of the ENSO, NAO, EA, EA/WR and SCA indices from the National Oceanic and Atmospheric Administration Climate Prediction Center (http://www.cpc.ncep.noaa.gov). We extracted 3-month average values from 1975–2013, corresponding to the months of January to March, February to April, March to May, April to June, May to July and June to August. These values represent six different LD before the start of the sugar beet harvesting season (September) in Europe^80^. For instance indices of climate variability at LD6 represent a 3-month average between January to March, therefore six months before the start of the sugar beet harvesting season (September).

We used the standardized Southern Oscillation Index (SOI) from 1951–2016, calculated from observed sea level pressure differences between Tahiti and Darwin (Australia), as a continuous measure of ENSO strength^81^. The time series of the four Northern Hemisphere teleconnection patterns from 1950–2016 represent monthly mean standardized 500-mb height anomalies at 20°N–90°N^82^. The NAO is the main mode of low-frequency variability over the North Atlantic, and consists of a north-south dipole of anomaly in surface pressure between Greenland and the central latitudes of the North Atlantic between 35°N and 40°N^82^. The EA pattern is the second prominent mode of low-frequency variability over the North Atlantic, consisting of a north-south dipole of anomaly centers that extends across the entire region from the East to the West. The EA/WR represents four main anomalies, centred over Europe and northern China, central North Atlantic and north of the Caspian Sea, while the SCA shows anomalies mainly in Scandinavia and western Russia^82^. The Euro-Atlantic region is mainly dominated by these four Northern Hemisphere teleconnections^11,35^.

### Index of agricultural impact

We obtained annual historical records of sugar beet production (in 1000 ton of fresh weight) from 1975 to 2013 for 232 NUTS2 from EUROSTAT. We examined sugar beet records for three major reasons: the European Union (EU) is the world’s leading producer of sugar beet^83^; sugar beet production is wide-spread within the EU territory; and generally, this crop is not extensively irrigated, thus having a strong dependency on rainfall^58^. We performed the analysis on historical records of sugar beet production instead of sugar beet yield (tons/hectare) due to the large unavailability of datasets of the latter in the vast majority of NUTS2 regions.

Years without sugar beet production records (M_N2_) at the NUTS2 level were filled according to the following method. First, we compared sugar beet production data registered at the national level (P_N_) by EUROSTAT (if available) with the sum registered at the NUTS2 level (P_N2_) for a given country (*c*) and year (*t*).

1 $${{{\rm{M}}}_{{\rm{N}}2,{\rm{t}},{\rm{c}}}={P}_{N,t,c}-\sum }^{}{{\rm{P}}}_{\mathrm{N2},t,c}$$

If there was a positive difference between both datasets $({{\rm{P}}}_{{\rm{N}},{\rm{t}},{\rm{c}}} > {\sum }^{}{{\rm{P}}}_{\mathrm{N2},t,c})$ and more than one NUTS2 region without a record, the missing production record at the NUTS2 (M_PN2_) region was filled proportionally with respect to the sum of its sugar beet harvesting area (HA_N2_):

2 $${{\rm{M}}}_{{\rm{PN}}2,{\rm{t}},{\rm{c}}}={{\rm{M}}}_{N2,t,c}/\frac{100}{{\sum }^{}H{A}_{N2}}$$

We assumed a positive and direct relationship between harvesting area and production: the larger the harvesting area, the higher the production, even though other factors, such as agricultural management, could affect production. Estimates of sugar beet harvesting areas are obtained from MIRCA 2000 and described in Sack *et al*. 2010^80^ (available in Fig. S5). In case of missing records in the NUTS2 region but no difference in the overall production $({{\rm{P}}}_{{\rm{N}},{\rm{t}},{\rm{c}}}={\sum }^{}{{\rm{P}}}_{{\rm{N}}2,{\rm{t}}})$, we assigned M_PN2,t,c_ = 0. Last, in the case of a negative difference $({{\rm{P}}}_{{\rm{N}},{\rm{t}},{\rm{c}}} < {\sum }^{}{{\rm{P}}}_{{\rm{N}}2,{\rm{t}}})$, the missing sugar beet production remained unaltered.

For each NUTS2 region, we calculated sugar beet production anomalies (observed value minus the multiyear mean) after removal of the linear trend (if the p-value is less than or equal to 0.1). We classified below zero anomalies as “low production”, and above zero anomalies as “high production”, creating a discrete agricultural impact indicator. Only NUTS2 regions with time series longer than 20 years were further investigated (NUTS2 areas that fit the criteria, and their respective summary of statistics are displayed in supplementary material Fig. S5 and Table S6, consecutively). In total, we were able to fit a FFT model in 207 NUTS2 regions.

### Statistical approach: Fast-and-Frugal Decision Tree (FFT)

In this study, we used FFT to predict sugar beet production as a function of indices of climate variability. In heuristic decision-making, FFT are simple decision trees for classifying cases (e.g. sugar beet production) into one of two classes (e.g. low production vs. high production) based on a particular predictor, or *cue*. FFT models establish simple rules for making decisions based on fast-and-frugal heuristics approach^84,85^, and offer simple and transparent search rules for practical decision problems^79^ as a competitive alternative for more complex Machine Learning and regression methods^86^.

As displayed in Fig. 5 step 3, the structure of an FFT determines the exact number, sequence and threshold of predictors that are applied to reach a final classification^84,87^. The FFTs algorithm applied in this study is limited to maximum five cues^79^. Consequently, a five-cue decision tree is based on the best five performing indices of climate variability. However, FFTs can be based on 1 to 5 cues since it uses non-compensatory decision rules, which apply a limited subset of all predictors for establishing a binary classification^86^. Non-compensatory algorithms ignore information, once a decision is completed, and therefore, no additional predictors can change such decision^79^. This aspect is often perceived to have both practical and statistical advantages over compensatory algorithms, such as regression models^86^. First, because by only using a partial subset of predictors, FFTs are relatively simple, and can perform better in predicting new data, thus they tend to avoid overfitting^44^. Second, FFT algorithms uses search rules that specify where to look for information and when to end search, which can guide decision makers in gathering information and assist in supporting decision tasks^44,85^.

The FFT algorithm is designed to learn from and make predictions on data. FFTs are fitted to a training dataset, which is used for learning the model, and deriving its parameter. In summary, the FFT’s algorithm is constructed as instructed: a) select predictors; b) determine a decision threshold for each predictor; c) determine the order of predictors; and d) determine the exit for each predictor^86^. By definition, FFTs must have either a negative or a positive exit (or both in the case of the final node of a decision tree)^79^.

The accuracy of the FFT is measured by the BACC Index, which is calculated based on the amount of correct decisions (Table 1) obtained from the ifan decision algorithm, which is described in detail in previous research^79^. In summary, the ifan decision algorithm tests several different thresholds of the investigated indices of climate variability to find one that maximizes the predictor’s accuracy. Consequently, once the set of multiple FFTs has been created, ifan selects the decision tree with the highest balanced accuracy.

**Table 1:** Definition of Standard Classification Statistics.

Standard Classification Statistics	Definition	Abbreviation	Formula
Hit Rate	Probability of a “True Low Production” (TL) over the total samples of “Low production” (LP).	HR	$(\frac{TL}{LP})\times 100$
Correct Rejections Rate	Probability of a “True High Production” (TH) over the total samples of “High Production” (HP)	CR	$(\frac{TH}{HP})\times 100$
False Alarm Rate	Probability of a false “Low Production”	FAR	1−CR
Miss Rate	Probability of a false “High Production”	MS	1−HR
Positive Predictive Value	Probability of a “True Low Production” over all “Low Production”	PPV	$\frac{{\rm{HR}}}{{\rm{HR}}+{\rm{FAR}}}$
Negative Predictive Value	Probability of a “True High Production” over all “High Production”	NPV	$\frac{{\rm{CR}}}{{\rm{CR}}+{\rm{MS}}}$
Balanced Accuracy	Average of Hit Rate and Correct Rejection	BACC	${\rm{HR}}\times 0,5+{\rm{CR}}\times 0,5$

In order to avoid overfitting, we cross-validated the FFTs using the train-test split method. For this, we partitioned 70% of all data for training the FFT models, and the other 30% for testing the models. We chose this validation method to assess the FFT potential success (if applied in a practical case), and its hindcast skill in predicting past sugar beet production events. In addition, this method takes less computation power than other cross-validation techniques such as k-fold. A more detailed explanation of the train-test split method is available in the Supplementary Material S8. For each FFT, we assessed the skill of the model to predict classes of “low production” and “high production” using the AUC index^88^. AUC measures how well the FFT can distinguish binary classes (low/high)^89^, displayed in supplementary material Fig. S9. The AUC index was calculated using the trapezoidal rule, and values can vary between 0 and 1, where a perfect prediction has an AUC = 1.0, and predictions that are randomly drawn are presumed to provide an AUC = 0.5^90^. We tested the statistical significance of the AUC by bootstrapping the values of the high and low sugar beet production events at the NUTS2 level using 1000 iterations (more details in the supplementary material 10). Field significance of the results was assessed using the binomial distribution^91^.

For NUTS2 regions with an AUC > 0.7, we display standard classification statistics such as HR, FAR, CR, MS, PPV and NPV. Their definition and formula are given in Table 1. We obtained FFT for each particular lead time and NUTS2 region through the following steps:

1. Calculating the pruning parameter of the model, meaning that we assessed the ideal size of decision trees by cross-validating the FFT models using train-test split method (see supplementary material S8);
2. Selecting the pruning parameter and decision tree that maximizes the BACC index of the tested model;
3. Calculating the AUC index for each best performing decision tree;
4. Assessing the statistical significance of the AUC obtained in step 3 by bootstrapping and calculating field significance (see supplementary material S10);
5. Analysing the performance of the significant FFT model by calculating standard classification statistics (Table 1).

After following such steps, we built a new FFT after recombining the two samples (training and testing) and adopting the pruning parameter that was found to maximize the BACC index. Hence, this procedure enables us to find the set of predictors which are most important for each NUTS2 region and lead time (Fig. 5 step 3). Since we aim at identifying regions where a robust model can be established based on the indices of atmospheric oscillation, we focus our results in regions with AUC > 0.7.

## Supplementary information

Supplementary Material

## References

[1] Godfray HCJ (2010). Food security: The challenge of feeding 9 billion people. Science.

[2] Pingali P (2007). Westernization of Asian diets and the transformation of food systems: Implications for research and policy. Food Policy.

[3] Ray, D. K., Mueller, N. D., West, P. C. & Foley, J. A. Yield Trends Are Insufficient to Double Global Crop Production by 2050. *PLoS One* **8** (2013). 10.1371/journal.pone.0066428 PMC3686737 23840465

[4] FAO. Achieving sustainable gains in agriculture. at, http://www.fao.org/docrep/014/am859e/am859e01.pdf. *FAO- South Am*. (2000).

[5] Bussay A, van der Velde M, Fumagalli D, Seguini L (2015). Improving operational maize yield forecasting in Hungary. Agric. Syst..

[6] Haigh T (2015). Climate Risk Management Mapping the decision points and climate information use of agricultural producers across the U. S. Corn Belt. Clim. Risk Manag..

[7] Iizumi T (2014). Impacts of El Niño Southern Oscillation on the global yields of major crops. Nat. Commun..

[8] Meinke H, Stone RC (2005). Seasonal and inter-annual climate forecasting: the new tool for increasing preparedness to climate variability and change in agricultural planning and operations. Clim. Change.

[9] Motha, R. P. & Baier, W. In *Increasing Climate Variability and Change*. 137–164 (Springer, 2005).

[10] Nnaji AO (2001). Forecasting seasonal rainfall for agricultural decision-making in northern Nigeria. Agric. For. Meteorol..

[11] Casanueva A, Rodríguez-Puebla C, Frías MD, González-Reviriego N (2014). Variability of extreme precipitation over Europe and its relationships with teleconnection patterns. Hydrol. Earth Syst. Sci..

[12] Sun X, Renard B, Thyer M, Westra S, Lang M (2015). A global analysis of the asymmetric effect of ENSO on extreme precipitation. J. Hydrol..

[13] UNMGCY. *Youth Science Policy Interface Publication – Special Edition: Disaster Risk Reduction: A Road of Opportunities*. at http://www.preventionweb.net/files/53923_53923unmgcydrreditionmay2017reduced.pdf. (2017).

[14] Veldkamp TIE, Eisner S, Wada Y, Aerts JCJH, Ward PJ (2015). Sensitivity of water scarcity events to ENSO-driven climate variability at the global scale. Hydrol. Earth Syst. Sci..

[15] Ward PJ, Beets W, Bouwer LM, Aerts JCJH, Renssen H (2010). Sensitivity of river discharge to ENSO. Geophys. Res. Lett..

[16] Ward PJ (2014). Strong influence of El Nino Southern Oscillation on flood risk around the world. Proc. Natl. Acad. Sci..

[17] Barlow M, Nigam S, Berbery EH (2001). ENSO, Pacific decadal variability, and U.S. summertime precipitation, drought, and stream flow. J. Clim..

[18] Dilley M, Heyman BN (1995). ENSO and disaster: droughts, floods and El Niño/Southern Oscillation warm events. Disasters.

[19] Donat MG (2014). Changes in extreme temperature and precipitation in the Arab region: Long-term trends and variability related to ENSO and NAO. Int. J. Climatol..

[20] Trenberth KE, Fasullo JT (2012). Climate extremes andclimate change: The Russian heat wave and other climate extremes of 2010. J. Geophys. Res. Atmos..

[21] Ferreyra RA (2001). A linked-modeling framework to estimate maize production risk associated with ENSO-related climate variability in Argentina. Agric. For. Meteorol..

[22] Ray DK, Gerber JS, MacDonald GK, West PC (2015). Climate variation explains a third of global crop yield variability. Nat. Commun..

[23] Rowhani P, Lobell DB, Linderman M, Ramankutty N (2011). Climate variability and crop production in Tanzania. Agric. For. Meteorol..

[24] Brönnimann, S. Impact of El Niño-Southern Oscillation on European climate. *Rev*. *Geophys*. **45** (2007).

[25] Guimarães Nobre G, Jongman B, Aerts J, Ward PJ (2017). The role of climate variability in extreme floods in Europe. Environ. Res. Lett..

[26] Lopez-Bustins JA, Martin-Vide J, Sanchez-Lorenzo A (2008). Iberia winter rainfall trends based upon changes in teleconnection and circulation patterns. Glob. Planet. Change.

[27] Mariotti A (2002). Euro-Mediterranean rainfall and ENSO—a seasonally varying relationship. Geophys. Res. Lett..

[28] Markovic D, Koch M (2014). Long-term variations and temporal scaling of hydroclimatic time series with focus on the German part of the Elbe River Basin. Hydrol. Process..

[29] Rios-Cornejo D, Penas A, Alvarez-Esteban R, del Rio S (2015). Links between teleconnection patterns and precipitation in Spain. Atmos. Res..

[30] Rodó X, Baert E, Comin FA (1997). Climate Dynamics Variations in seasonal rainfall in Southern Europe during the present century: relationships with the North Atlantic Oscillation and the. Clim. Dyn..

[31] Struglia MV, Mariotti A, Filograsso A (2004). River discharge into the Mediterranean sea: Climatology and aspects of the observed variability. J. Clim..

[32] Bueh C, Nakamura H (2007). Scandinavian pattern and its climatic impact. Q. J. R. Meteorol. Soc..

[33] Cantelaube P, Terres J, Doblas-Reyes FJ (2004). Influence of climate variability on European agriculture- analysis of winter wheat production. Clim. Res..

[34] Kettlewell PS, Stephenson DB, Atkinson MD, Hollins PD (2003). Summer rainfall and wheat grain quality: relationships with the North Atlantic Oscillation. Weather.

[35] Ceglar A, Turco M, Toreti A, Doblas-Reyes FJ (2017). Linking crop yield anomalies to large-scale atmospheric circulation in Europe. Agric. For. Meteorol..

[36] Gouveia, C. & Trigo, R. M. In *geoENV VI–Geostatistics for Environmental Applications*. 335–345 (Springer, 2008).

[37] Lorenzo MN, Taboada JJ, Lorenzo JF, Ramos AM (2013). Influence of climate on grape production and wine quality in the Rías Baixas, north-western Spain. Reg. Environ. Chang..

[38] Marta AD, Grifoni D, Mancini M (2011). The influence of climate on durum wheat quality in Tuscany, Central Italy. Int. J. Biometeorol..

[39] Gimeno L (2002). Identification of empirical relationships between indices of ENSO and NAO and agricultural yields in Spain. Clim. Res..

[40] Hollinger, S. E. Incorporating Weather and Climate data into Integrated Crop Management Systems. *Clim*. *Agric*. *Drought Misc*. *Pap*. 1 (1991).

[41] Calanca P, Bolius D, Weigel AP, Liniger MA (2011). Application of long-range weather forecasts to agricultural decision problems inEurope. J. Agric. Sci..

[42] Easterling WE, Mjelde JW (1987). The importance of seasonal climate prediction lead time in agricultural decision making. Agric. For. Meteorol..

[43] Mase AS, Prokopy LS (2014). Unrealized potential: A review of perceptions and use of weather and climate information in agricultural decision making. Weather. Clim. Soc..

[44] Phillips ND, Woike JK, Gaissmaier W (2017). FFTrees: A toolbox to create, visualize, and evaluate fast-and-frugal decision trees. Judgm. Decis. Mak..

[45] Hurrell JW, Deser C (2010). North Atlantic climate variability: The role of the North Atlantic Oscillation. J. Mar. Syst..

[46] Ionita M (2014). The impact of the East Atlantic/Western Russia pattern on the hydroclimatology of Europe from mid-winter to late spring. Climate.

[47] Gouveia C, Trigo RM, DaCamara CC, Libonati R, Pereira JMC (2008). The North Atlantic Oscillation and European vegetation dynamics. Int. J. Climatol..

[48] Kim M, Mccarl BA (2005). The Agricultural Value Of Information On The North Atlantic Oscillation: Yield And Economic Effects. Clim. Change.

[49] Heino, M. *et al*. Two-thirds of global cropland area impacted by climate oscillations. *Nat*. *Commun*. **9** (2018). 10.1038/s41467-017-02071-5 PMC5871885 29593219

[50] Gonsamo, A. & Chen, J. M. Winter teleconnections can predict the ensuing summer European crop productivity: Fig. 1. *Proc*. *Natl*. *Acad*. *Sci*. 10.1073/pnas.1503450112 (2015). 10.1073/pnas.1503450112 PMC4426402 25873740

[51] Res C (2002). Identification of empirical relationships between indices of ENSO and NAO and agricultural yields in Spain. Clim. Res..

[52] Kettlewell, P. S., Easey, J., Stephenson, D. B. & Poulton, P. R. Soil moisture mediates association between the winter North Atlantic Oscillation and summer growth in the Park Grass Experiment. *Proc*. *R*. *Soc*. *B Biol*. *Sci*., 10.1098/rspb.2005.3428 (2006). 10.1098/rspb.2005.3428 PMC1560263 16600894

[53] Wang, G., Dolman, A. J. & Alessandri, A. A summer climate regime over Europe modulated by the North Atlantic Oscillation. *Hydrol*. *Earth Syst*. *Sci*. 10.5194/hess-15-57-2011 (2011).

[54] Petkeviciene B, others (2009). The effects of climate factors on sugar beet early sowing timing. Agron. Res.

[55] Clarke, N., Hetschkun, H., Jones, C., Boswell, E. & Marfaing, H. In *Interacting stresses on plants in a changing climate* 511–524 (Springer, 1993).

[56] Gao T, Yu Jyi, Paek H (2017). Impacts of four northern-hemisphere teleconnection patterns on atmospheric circulations over Eurasia and the Pacific. Theor. Appl. Climatol..

[57] Shaman J (2014). The seasonal effects of ENSO on European precipitation: Observational analysis. J. Clim..

[58] EUROSTAT. Agri-environmental indicator - irrigation. at, http://ec.europa.eu/eurostat/statistics-explained/index.php/File:Irrigated_area_of_intensive_crops_(potatoes_and_sugar_beet),_2010_(%25_of_total_area_of_each_crop).png. (2010).

[59] Wriedt, G., der Velde, M., Aloe, A. & Bouraoui, F. Water requirements for irrigation in the European Union. *EUR-Scientific Tech*. *Res*. *Reports*, *EUR* **23453** (2008).

[60] Wriedt, G., Van der Velde, M., Aloe, A. & Bouraoui, F. Estimating irrigation water requirements in Europe. *J*. *Hydrol*, 10.1016/j.jhydrol.2009.05.018 (2009).

[61] FAO. Crop Water Information: Sugarbeet. at http://web.archive.org/web/20160831112022/http://www.fao.org/nr/water/cropinfo_sugarbeet.html. (2015).

[62] Baruth B, Royer A, Klisch A, Genovese G (2008). The use of remote sensing within the MARS crop yield monitoring system of the European Commission. Proc. ISPRS.

[63] van der Velde, M. *et al*. Use and relevance of European Union crop monitoring and yield forecasts. *Agric*. *Syst* (2018).

[64] Lecerf, R., Ceglar, A., López-Lozano, R., Van Der Velde, M. & Baruth, B. Assessing the information in crop model and meteorological indicators to forecast crop yield overEurope. *Agric*. *Syst* (2018).

[65] European Comission. EU sugar policy. at, https://ec.europa.eu/agriculture/sugar_en (2017).

[66] Romano A (2012). Morpho-physiological responses of sugar beet (Beta vulgaris L.) genotypes to drought stress. Acta Physiol. Plant..

[67] Pidgeon JD (2001). Climatic impact on the productivity of sugar beet inEurope, 1961-1995. Agric. For. Meteorol..

[68] Snyder, R. L. & de Melo-Abreu, J. P. *Frost protection: fundamentals*, *practice and economics*. (Food and agriculture organization of the United Nations, 2005).

[69] European Parliament and Council. *Directive on the taking-up and pursuit of the business of insurance and reinsurance (Solvency II)*, *Directive2009/138/EC* (2009).

[70] Ober E (2001). The search for drought tolerance insugar beet. Br. sugar beet Rev..

[71] Van Swaaij A, Heijbroek W, Basting JL (2001). Testing and improving seed vigour in sugar beet. Int. sugar J..

[72] Vandendriessche J (1995). Crop Models and Decision Support Systems for Yield Forecasting and Management of the Sugar Beet Crop. Eur. J. Agron..

[73] Kenter C, Hoffmann CM, Märländer B (2006). Effects of weather variables on sugar beet yield development (Beta vulgaris L.). Eur. J. Agron..

[74] JRC MARS Bulletin. *Crop monitoring in Europe*, *November 2016: Arrival of the first frosts*. **24** (2016).

[75] Dunstone N (2016). Skilful predictions of the winter North Atlantic Oscillation one year ahead. Nat. Geosci..

[76] Iglesias I, Lorenzo MN, Taboada JJ (2014). Seasonal predictability of the east atlantic pattern from sea surface temperatures. PLoS One.

[77] Gonzalez PLM, Goddard L (2016). Long-lead ENSO predictability from CMIP5 decadal hindcasts. Clim. Dyn..

[78] Ossó, A., Sutton, R., Shaffrey, L. & Dong, B. Observational evidence of European summer weather patterns predictable from spring. *Proc*. *Natl*. *Acad*. *Sci*. 201713146 10.1073/pnas.1713146114 (2017). 10.1073/pnas.1713146114 PMC5776804 29255052

[79] Phillips ND, Neth H, Woike JK, Gaissmaier W (2017). FFTrees: A toolbox to create, visualize, and evaluate fast-and-frugal decision trees. Judgm. Decis. Mak..

[80] Sacks WJ, Deryng D, Foley JA (2010). Crop planting dates: an analysis of global patterns. Glob. Ecol. Biogeogr..

[81] NOAA. The Southern Oscillation Index. at, http://www.cpc.ncep.noaa.gov/products/analysis_monitoring/ensocycle/soi.shtml. (2005).

[82] Barnston AG, Livezey RE (1987). Classification, Seasonality and Persistence of Low-Frequency Atmospheric Circulation Patterns. Mon. Weather Rev..

[83] EUROSTAT. Agricultural production - crops. at, http://ec.europa.eu/eurostat/statistics-explained/index.php/Agricultural_production_-_crops#Sugar_beet (2016).

[84] Gigerenzer, G., Czerlinski, J. & Martignon, L. How good are fast and frugal heuristics? *Decis*. *Sci*. *Technol*. 81–103 (1999).

[85] Raab, M. & Gigerenzer, G. The power of simplicity: A fast-and-frugal heuristics approach to performance science. *Front*. *Psychol*. 10.3389/fpsyg.2015.01672 (2015). 10.3389/fpsyg.2015.01672 PMC4625029 26579051

[86] Nobre, G. G. *et al*. Financing agricultural drought risk through ex-ante cash transfers. *Sci*. *Total Environ* (2018). 10.1016/j.scitotenv.2018.10.406 30414582

[87] Gigerenzer, G. & Todd, P. M. In *Simple heuristics that make us smart* 3–34 (Oxford University Press (1999).

[88] Metz CE (1978). Basic principles of ROC analysis. Semin. Nucl. Med..

[89] Zweig H, Mark, Campbell G (1993). Receiver-Operating Clinical Medicine (ROC) Plots: A Fundamental Evaluation Tool.

[90] Hamill TM, Juras J (2006). Measuring forecast skill: is it real skill or is it the varying climatology?. Q. J. R. Meteorol. Soc..

[91] Livezey RE, Chen WY (1983). Statistical Field Significance and its Determination by Monte Carlo Techniques. Monthly Weather Review.

