# Supplementary material for: Translating large-scale climate variability into crop production forecast in Europe
Source: Sci Rep. 2019 Feb 4;9:1277. doi: 10.1038/s41598-018-38091-4 (PMC6361969; doi:10.1038/s41598-018-38091-4)
Supplement: Supplementary file 1 — Supplementary Material [file 41598_2018_38091_MOESM1_ESM.docx]

Scientific Reports

Supplementary Material for

**Translating large-scale climate variability into crop production forecast in Europe**

Gabriela Guimarães Nobre^1^,*, Johannes E Hunink^2^ , Bettina Baruth^3^, Jeroen C J H Aerts^1^ and Philip J Ward^1^

^1^ Institute for Environmental Studies (IVM), Vrije Universiteit Amsterdam, De Boelelaan 1087, 1081 HV Amsterdam, The Netherlands
^2^ FutureWater, Spain
^3^ Directorate Sustainable Resources, European Commission, Joint Research Centre

*Corresponding e-mail: [g.guimaraesnobre@vu.nl](mailto:g.guimaraesnobre@vu.nl)

S1 Standard classification statistics for areas with predictive skill (AUC>0.7)

Table S1.1. Standard classification statistics for best performing FFT at lead time 6.

| **NUTS2 ID** | **AUC** | **False Alarm (%)** | **Miss (%)** | **Correct Rejection (%)** | **Hit Rate (%)** | **Negative Predictive Value (%)** | **Positive Predictive Value (%)** | **Cue(s)** | **Number of Predictors** | **Number of Missing Values at the NUTS2 Level** |
| --- | --- | --- | --- | --- | --- | --- | --- | --- | --- | --- |
| AT13 | 0,75 | 33 | 17 | 67 | 83 | 71 | 80 | NAO;SOI;EA | 3 | 0 |
| AT21 | 0,80 | 0 | 40 | 100 | 60 | 100 | 78 | SOI;EA;NAO;EAWR;SCA | 5 | 0 |
| AT22 | 0,75 | 50 | 0 | 50 | 100 | 67 | 100 | EA;SCA;NAO | 3 | 0 |
| AT31 | 0,73 | 40 | 14 | 60 | 86 | 75 | 75 | EA | 1 | 0 |
| BE22 | 0,70 | 60 | 0 | 40 | 100 | 70 | 100 | SCA;EA;EAWR | 3 | 0 |
| BE34 | 1,00 | 0 | 0 | 100 | 100 | 100 | 100 | EAWR;NAO;SOI;EA | 4 | 0 |
| CZ01 | 0,80 | 0 | 40 | 100 | 60 | 100 | 60 | EAWR | 1 | 39 |
| CZ02 | 0,80 | 0 | 40 | 100 | 60 | 100 | 60 | EAWR | 1 | 39 |
| CZ03 | 0,80 | 0 | 40 | 100 | 60 | 100 | 60 | EAWR | 1 | 39 |
| CZ04 | 0,80 | 0 | 40 | 100 | 60 | 100 | 60 | EAWR | 1 | 39 |
| CZ05 | 0,80 | 0 | 40 | 100 | 60 | 100 | 60 | EAWR | 1 | 39 |
| CZ06 | 0,80 | 0 | 40 | 100 | 60 | 100 | 60 | EAWR | 1 | 39 |
| CZ07 | 0,80 | 0 | 40 | 100 | 60 | 100 | 60 | EAWR | 1 | 39 |
| CZ08 | 0,80 | 0 | 40 | 100 | 60 | 100 | 60 | EAWR | 1 | 39 |
| DE14 | 0,80 | 0 | 40 | 100 | 60 | 100 | 78 | NAO;SCA | 2 | 31 |
| DE22 | 0,71 | 0 | 57 | 100 | 43 | 100 | 56 | SCA;EAWR;SOI;NAO | 4 | 31 |
| DE24 | 0,80 | 0 | 40 | 100 | 60 | 100 | 78 | NAO;SCA | 2 | 31 |
| DE27 | 0,71 | 0 | 57 | 100 | 43 | 100 | 56 | SCA;EAWR;SOI;NAO | 4 | 31 |
| DE50 | 0,78 | 11 | 33 | 89 | 67 | 67 | 89 | EAWR;SCA;NAO | 3 | 18 |
| DE72 | 0,75 | 0 | 50 | 100 | 50 | 100 | 50 | SCA;NAO;EAWR | 3 | 33 |
| DE94 | 0,70 | 0 | 60 | 100 | 40 | 100 | 70 | NAO | 1 | 32 |
| DEB2 | 0,70 | 0 | 60 | 100 | 40 | 100 | 70 | NAO | 1 | 31 |
| DEF0 | 0,70 | 60 | 0 | 40 | 100 | 70 | 100 | EAWR;NAO;EA | 3 | 9 |
| DEG0 | 0,76 | 20 | 29 | 80 | 71 | 83 | 67 | EA | 1 | 24 |
| FR23 | 0,86 | 0 | 29 | 100 | 71 | 100 | 71 | NAO;SCA;EAWR;EA | 4 | 2 |
| FR25 | 0,75 | 0 | 50 | 100 | 50 | 100 | 67 | EA;EAWR;SOI;NAO | 4 | 2 |
| FR26 | 0,70 | 0 | 60 | 100 | 40 | 100 | 70 | SOI;EA;SCA;EAWR;NAO | 5 | 2 |
| HU23 | 0,75 | 25 | 25 | 75 | 75 | 75 | 75 | NAO;SCA;SOI;EA;EAWR | 5 | 20 |
| HU31 | 0,75 | 25 | 25 | 75 | 75 | 75 | 75 | EA;NAO;SCA | 3 | 20 |
| HU32 | 0,75 | 50 | 0 | 50 | 100 | 86 | 100 | NAO;SOI;EA | 3 | 20 |
| IE01 | 0,81 | 25 | 12 | 75 | 88 | 88 | 75 | NAO;SCA;EA;EAWR;SOI | 5 | 38 |
| IE02 | 0,81 | 25 | 12 | 75 | 88 | 88 | 75 | NAO;SCA;EA;EAWR;SOI | 5 | 38 |
| ITC4 | 0,70 | 50 | 10 | 50 | 90 | 90 | 50 | NAO;SCA;SOI | 3 | 6 |
| ITF1 | 0,83 | 20 | 14 | 80 | 86 | 86 | 80 | EAWR;SCA | 2 | 6 |
| ITH1 | 0,83 | 20 | 14 | 80 | 86 | 86 | 80 | NAO;SCA;EA;EAWR | 4 | 32 |
| ITH2 | 0,83 | 20 | 14 | 80 | 86 | 86 | 80 | NAO;SCA;EA;EAWR | 4 | 32 |
| ITI1 | 0,80 | 0 | 40 | 100 | 60 | 100 | 33 | EAWR;NAO;SCA | 3 | 6 |
| ITI4 | 0,70 | 50 | 10 | 50 | 90 | 90 | 50 | NAO;SCA;SOI | 3 | 6 |
| NL22 | 0,81 | 25 | 12 | 75 | 88 | 88 | 75 | EA;NAO | 2 | 6 |
| NL33 | 0,75 | 50 | 0 | 50 | 100 | 80 | 100 | NAO;EA;EAWR | 3 | 1 |
| NL41 | 0,70 | 50 | 10 | 50 | 90 | 90 | 50 | EAWR;NAO | 2 | 1 |
| NL42 | 0,86 | 0 | 29 | 100 | 71 | 100 | 71 | NAO;EAWR;SCA;EA | 4 | 1 |
| PL33 | 1,00 | 0 | 0 | 100 | 100 | 100 | 100 | EAWR;NAO;SCA;EA | 4 | 20 |
| PL34 | 0,75 | 50 | 0 | 50 | 100 | 86 | 100 | EA;SCA | 2 | 20 |
| PL43 | 0,75 | 50 | 0 | 50 | 100 | 86 | 100 | EA | 1 | 20 |
| PL63 | 0,90 | 0 | 20 | 100 | 80 | 100 | 75 | SOI;EAWR;EA;NAO | 4 | 20 |
| PT20 | 0,81 | 25 | 12 | 75 | 88 | 88 | 75 | EA;EAWR;SOI | 3 | 6 |
| RO21 | 0,79 | 43 | 0 | 57 | 100 | 25 | 100 | EAWR;NAO;SCA;SOI;EA | 5 | 20 |
| RO32 | 0,75 | 0 | 50 | 100 | 50 | 100 | 86 | SCA;SOI | 2 | 25 |
| RO41 | 0,75 | 0 | 50 | 100 | 50 | 100 | 86 | EA;SCA | 2 | 24 |
| SE22 | 0,86 | 0 | 29 | 100 | 71 | 100 | 50 | NAO;SCA;SOI | 3 | 18 |
| SK02 | 0,90 | 0 | 20 | 100 | 80 | 100 | 75 | EAWR;NAO | 2 | 20 |
| SK03 | 0,75 | 50 | 0 | 50 | 100 | 86 | 100 | SOI;EAWR;NAO;EA; | 4 | 20 |
| SK04 | 0,70 | 0 | 60 | 100 | 40 | 100 | 50 | SOI | 1 | 22 |

Table S1.2. Standard classification statistics for best performing FFT at lead time 5.

| **NUTS2 ID** | **AUC** | **False Alarm (%)** | **Miss (%)** | **Correct Rejection (%)** | **Hit Rate (%)** | **Negative Predictive Value (%)** | **Positive Predictive Value (%)** | **Cue(s)** | **Number of Predictors** | **Number of Missing Values at the NUTS2 Level** |
| --- | --- | --- | --- | --- | --- | --- | --- | --- | --- | --- |
| AT22 | 0,75 | 50 | 0 | 50 | 100 | 100 | 67 | EA | 1 | 0 |
| AT31 | 0,80 | 40 | 0 | 60 | 100 | 100 | 78 | EA | 1 | 0 |
| BE34 | 0,75 | 17 | 33 | 83 | 67 | 71 | 80 | SOI;EA | 2 | 0 |
| DE13 | 0,75 | 50 | 0 | 50 | 100 | 100 | 67 | NAO | 1 | 31 |
| DE14 | 0,83 | 14 | 20 | 86 | 80 | 86 | 80 | EA;SOI;NAO | 3 | 31 |
| DE21 | 0,88 | 25 | 0 | 75 | 100 | 100 | 89 | SCA;NAO | 2 | 31 |
| DE24 | 0,83 | 14 | 20 | 86 | 80 | 86 | 80 | EA;SOI;NAO | 3 | 31 |
| DE25 | 0,75 | 50 | 0 | 50 | 100 | 100 | 67 | NAO | 1 | 31 |
| DE40 | 0,81 | 0 | 38 | 100 | 62 | 57 | 100 | EA;SOI;NAO | 3 | 39 |
| DE50 | 0,72 | 22 | 33 | 78 | 67 | 88 | 50 | NAO;EA;EAWR;SOI | 4 | 18 |
| DE71 | 0,72 | 33 | 22 | 67 | 78 | 50 | 88 | NAO;SOI;EAWR | 3 | 33 |
| DE73 | 0,72 | 33 | 22 | 67 | 78 | 50 | 88 | NAO;EAWR | 2 | 33 |
| DE80 | 0,83 | 20 | 14 | 80 | 86 | 80 | 86 | NAO;EA | 2 | 24 |
| DE91 | 0,81 | 25 | 12 | 75 | 88 | 75 | 88 | NAO;EAWR | 2 | 32 |
| DE93 | 0,88 | 25 | 0 | 75 | 100 | 100 | 89 | NAO | 1 | 32 |
| DE94 | 0,83 | 14 | 20 | 86 | 80 | 86 | 80 | EA;SOI;NAO | 3 | 32 |
| DEA3 | 0,75 | 50 | 0 | 50 | 100 | 100 | 67 | NAO | 1 | 31 |
| DEA4 | 0,88 | 25 | 0 | 75 | 100 | 100 | 89 | SCA;NAO | 2 | 31 |
| DEA5 | 0,75 | 17 | 33 | 83 | 67 | 71 | 80 | EAWR;SOI;SCA;NAO | 4 | 31 |
| DEB1 | 0,71 | 57 | 0 | 43 | 100 | 100 | 56 | SCA;NAO | 2 | 31 |
| DEC0 | 0,81 | 0 | 38 | 100 | 62 | 57 | 100 | EA;SOI;NAO | 3 | 39 |
| DED4 | 0,81 | 0 | 38 | 100 | 62 | 57 | 100 | EA;SOI;NAO | 3 | 39 |
| DED5 | 0,81 | 0 | 38 | 100 | 62 | 57 | 100 | EA;SOI;NAO | 3 | 39 |
| DEE0 | 0,81 | 0 | 38 | 100 | 62 | 57 | 100 | EA;SOI;NAO | 3 | 39 |
| DEF0 | 0,73 | 40 | 14 | 60 | 86 | 75 | 75 | EA | 1 | 9 |
| DEG0 | 0,80 | 40 | 0 | 60 | 100 | 100 | 78 | EA;SCA;NAO | 3 | 24 |
| FR10 | 0,75 | 50 | 0 | 50 | 100 | 100 | 67 | EA | 1 | 2 |
| FR22 | 0,75 | 50 | 0 | 50 | 100 | 100 | 67 | EA | 1 | 2 |
| FR23 | 0,79 | 0 | 43 | 100 | 57 | 62 | 100 | NAO | 1 | 2 |
| FR25 | 0,75 | 50 | 0 | 50 | 100 | 100 | 67 | EA | 1 | 2 |
| FR30 | 0,94 | 0 | 12 | 100 | 88 | 80 | 100 | NAO;EA;EAWR;SCA;SOI | 5 | 2 |
| FR51 | 0,75 | 50 | 0 | 50 | 100 | 100 | 67 | EA | 1 | 2 |
| HU33 | 0,75 | 50 | 0 | 50 | 100 | 100 | 86 | EAWR | 1 | 20 |
| IE01 | 0,75 | 25 | 25 | 75 | 75 | 60 | 86 | NAO;EA | 2 | 38 |
| IE02 | 0,75 | 25 | 25 | 75 | 75 | 60 | 86 | NAO;EA | 2 | 38 |
| ITF1 | 0,70 | 60 | 0 | 40 | 100 | 100 | 70 | SOI | 1 | 6 |
| ITF6 | 0,75 | 50 | 0 | 50 | 100 | 100 | 91 | SCA | 1 | 6 |
| ITH1 | 0,76 | 20 | 29 | 80 | 71 | 67 | 83 | NAO;EA;EAWR | 3 | 32 |
| ITH2 | 0,76 | 20 | 29 | 80 | 71 | 67 | 83 | NAO;EA;EAWR;; | 3 | 32 |
| NL22 | 0,94 | 0 | 12 | 100 | 88 | 80 | 100 | EA;NAO;EAWR;SOI;SCA | 5 | 6 |
| NL42 | 0,73 | 40 | 14 | 60 | 86 | 75 | 75 | NAO;EAWR | 2 | 1 |
| PL32 | 0,83 | 0 | 33 | 100 | 67 | 83 | 100 | EAWR;SOI;EA | 3 | 20 |
| PL33 | 0,80 | 0 | 40 | 100 | 60 | 60 | 100 | EAWR | 1 | 20 |
| PL43 | 0,83 | 0 | 33 | 100 | 67 | 50 | 100 | EA;EAWR | 2 | 20 |
| PL61 | 0,75 | 50 | 0 | 50 | 100 | 100 | 67 | SOI;NAO | 2 | 20 |
| SK02 | 0,80 | 0 | 40 | 100 | 60 | 60 | 100 | EA;EAWR;SOI | 3 | 20 |
| SK04 | 0,83 | 33 | 0 | 67 | 100 | 100 | 83 | SOI;EA;SCA | 3 | 22 |

Table S1.3. Standard classification statistics for best performing FFT at lead time 4.

| **NUTS2 ID** | **AUC** | **False Alarm (%)** | **Miss (%)** | **Correct Rejection (%)** | **Hit Rate (%)** | **Negative Predictive Value (%)** | **Positive Predictive Value (%)** | **Cue(s)** | **Number of Predictors** | **Number of Missing Values at the NUTS2 Level** |
| --- | --- | --- | --- | --- | --- | --- | --- | --- | --- | --- |
| AT12 | 0,76 | 29 | 20 | 71 | 80 | 83 | 67 | EA;NAO | 2 | 0 |
| AT22 | 0,75 | 50 | 0 | 50 | 100 | 100 | 67 | EAWR;EA | 2 | 0 |
| AT31 | 0,76 | 20 | 29 | 80 | 71 | 67 | 83 | EA;EAWR;SOI;SCA | 4 | 0 |
| DE13 | 0,92 | 0 | 17 | 100 | 83 | 86 | 100 | EAWR;SCA;SOI;NAO | 4 | 31 |
| DE25 | 0,92 | 0 | 17 | 100 | 83 | 86 | 100 | SCA;EA;EAWR;SOI;NAO | 5 | 31 |
| DE80 | 0,76 | 20 | 29 | 80 | 71 | 67 | 83 | EA;SOI;EAWR;SCA;NAO | 5 | 24 |
| FI20 | 0,75 | 33 | 17 | 67 | 83 | 67 | 83 | NAO;EAWR | 2 | 20 |
| FR10 | 0,83 | 0 | 33 | 100 | 67 | 75 | 100 | EA | 1 | 2 |
| FR21 | 0,75 | 25 | 25 | 75 | 75 | 60 | 86 | SCA;EAWR;EA | 3 | 2 |
| FR23 | 0,73 | 40 | 14 | 60 | 86 | 75 | 75 | EA;EAWR;SOI;SCA | 4 | 2 |
| FR30 | 0,81 | 0 | 38 | 100 | 62 | 57 | 100 | EAWR;SCA;EA | 3 | 2 |
| FR41 | 0,78 | 33 | 11 | 67 | 89 | 67 | 89 | EAWR;NAO | 2 | 2 |
| FR92 | 0,75 | 50 | 0 | 50 | 100 | 100 | 78 | EAWR;SOI;NAO;EA | 4 | 5 |
| HU21 | 0,83 | 0 | 33 | 100 | 67 | 83 | 100 | EAWR;SCA | 2 | 20 |
| HU23 | 0,75 | 50 | 0 | 50 | 100 | 100 | 67 | SCA;EA;EAWR | 3 | 20 |
| HU32 | 0,83 | 0 | 33 | 100 | 67 | 50 | 100 | SCA;SOI | 2 | 20 |
| ITF4 | 0,75 | 17 | 33 | 83 | 67 | 71 | 80 | SCA;EAWR;EA;NAO | 4 | 6 |
| ITI1 | 0,70 | 50 | 10 | 50 | 90 | 50 | 90 | EAWR;SCA;SOI | 3 | 6 |
| NL11 | 0,80 | 0 | 40 | 100 | 60 | 33 | 100 | SCA;NAO | 2 | 1 |
| NL13 | 0,73 | 14 | 40 | 86 | 60 | 75 | 75 | NAO | 1 | 1 |
| NL34 | 0,83 | 17 | 17 | 83 | 83 | 83 | 83 | SCA;NAO | 2 | 1 |
| NL41 | 0,75 | 0 | 50 | 100 | 50 | 29 | 100 | NAO | 1 | 1 |
| PL32 | 0,83 | 0 | 33 | 100 | 67 | 83 | 100 | EAWR;SOI;EA | 3 | 20 |
| PL51 | 0,83 | 33 | 0 | 67 | 100 | 100 | 83 | EA;SCA | 2 | 20 |
| RO31 | 0,70 | 60 | 0 | 40 | 100 | 100 | 50 | EA;EAWR | 2 | 21 |

Table S1.4. Standard classification statistics for best performing FFT at lead time 3.

| **NUTS2 ID** | **AUC** | **False Alarm (%)** | **Miss (%)** | **Correct Rejection (%)** | **Hit Rate (%)** | **Negative Predictive Value (%)** | **Positive Predictive Value (%)** | **Cue(s)** | **Number of Predictors** | **Number of Missing Values at the NUTS2 Level** |
| --- | --- | --- | --- | --- | --- | --- | --- | --- | --- | --- |
| BE21 | 0,75 | 17 | 33 | 83 | 67 | 71 | 80 | SCA;EAWR;EA;NAO | 4 | 0 |
| BE22 | 0,76 | 20 | 29 | 80 | 71 | 67 | 83 | EAWR | 1 | 0 |
| BE23 | 0,75 | 17 | 33 | 83 | 67 | 71 | 80 | SCA;EAWR;EA;NAO | 4 | 0 |
| BE25 | 0,75 | 0 | 50 | 100 | 50 | 67 | 100 | EA;NAO;SCA;SOI;EAWR | 5 | 0 |
| BE34 | 0,75 | 50 | 0 | 50 | 100 | 100 | 67 | EA;NAO;EAWR;SCA;SOI | 5 | 0 |
| DE13 | 0,75 | 0 | 50 | 100 | 50 | 67 | 100 | EAWR;SCA | 2 | 31 |
| DE14 | 0,73 | 14 | 40 | 86 | 60 | 75 | 75 | EAWR | 1 | 31 |
| DE22 | 0,73 | 40 | 14 | 60 | 86 | 75 | 75 | SCA;NAO;SOI | 3 | 31 |
| DE23 | 0,73 | 40 | 14 | 60 | 86 | 75 | 75 | SCA;SOI | 2 | 31 |
| DE24 | 0,73 | 14 | 40 | 86 | 60 | 75 | 75 | EAWR | 1 | 31 |
| DE25 | 0,75 | 33 | 17 | 67 | 83 | 80 | 71 | SCA | 1 | 31 |
| DE27 | 0,73 | 40 | 14 | 60 | 86 | 75 | 75 | SCA;NAO;SOI | 3 | 31 |
| DE50 | 0,89 | 22 | 0 | 78 | 100 | 100 | 60 | SOI | 1 | 18 |
| DE91 | 0,75 | 50 | 0 | 50 | 100 | 100 | 80 | SCA;EAWR | 2 | 32 |
| DE92 | 0,75 | 50 | 0 | 50 | 100 | 100 | 80 | SCA;EAWR | 2 | 32 |
| DE94 | 0,73 | 14 | 40 | 86 | 60 | 75 | 75 | EAWR | 1 | 32 |
| DEA1 | 0,70 | 60 | 0 | 40 | 100 | 100 | 70 | SCA;SOI;EAWR | 3 | 31 |
| DEA2 | 0,70 | 60 | 0 | 40 | 100 | 100 | 70 | SCA;SOI;EAWR | 3 | 31 |
| DEA5 | 0,75 | 33 | 17 | 67 | 83 | 80 | 71 | SOI | 1 | 31 |
| DEB1 | 0,80 | 0 | 40 | 100 | 60 | 78 | 100 | SCA;EAWR | 2 | 31 |
| DEB2 | 0,73 | 14 | 40 | 86 | 60 | 75 | 75 | EAWR | 1 | 31 |
| DEG0 | 0,70 | 60 | 0 | 40 | 100 | 100 | 70 | SCA;EA | 2 | 24 |
| EL11 | 0,75 | 25 | 25 | 75 | 75 | 60 | 86 | SCA | 1 | 16 |
| EL14 | 0,75 | 0 | 50 | 100 | 50 | 50 | 100 | SOI;EAWR;EA;NAO;SCA | 5 | 11 |
| EL24 | 0,75 | 17 | 33 | 83 | 67 | 71 | 80 | SOI;EAWR;EA | 3 | 16 |
| ES22 | 0,81 | 38 | 0 | 62 | 100 | 100 | 57 | EA;SCA;NAO;SOI | 4 | 3 |
| FR24 | 0,71 | 0 | 57 | 100 | 43 | 56 | 100 | SOI;EA | 2 | 2 |
| FR71 | 0,70 | 50 | 10 | 50 | 90 | 50 | 90 | SOI;NAO | 2 | 2 |
| FR92 | 0,88 | 25 | 0 | 75 | 100 | 100 | 88 | SOI;EA;EAWR | 3 | 5 |
| FR93 | 0,75 | 50 | 0 | 50 | 100 | 100 | 78 | EA;EAWR;NAO;SCA | 4 | 5 |
| HU21 | 1,00 | 0 | 0 | 100 | 100 | 100 | 100 | EA;EAWR | 2 | 20 |
| HU23 | 0,75 | 50 | 0 | 50 | 100 | 100 | 67 | NAO;EA;EAWR | 3 | 20 |
| IE01 | 0,94 | 0 | 12 | 100 | 88 | 80 | 100 | SOI | 1 | 38 |
| IE02 | 0,94 | 0 | 12 | 100 | 88 | 80 | 100 | SOI | 1 | 38 |
| ITF6 | 0,90 | 0 | 20 | 100 | 80 | 50 | 100 | SOI | 1 | 6 |
| ITH1 | 0,70 | 60 | 0 | 40 | 100 | 100 | 70 | NAO;EA;EAWR;SCA;SOI | 5 | 32 |
| ITH2 | 0,70 | 60 | 0 | 40 | 100 | 100 | 70 | NAO;EA;EAWR;SCA;SOI | 5 | 32 |
| ITH5 | 0,72 | 33 | 22 | 67 | 78 | 50 | 88 | EA;NAO;SCA;EAWR | 4 | 39 |
| ITI3 | 0,72 | 33 | 22 | 67 | 78 | 50 | 88 | EA;NAO;SCA;EAWR | 4 | 39 |
| NL12 | 0,81 | 25 | 12 | 75 | 88 | 75 | 88 | EAWR;SCA | 2 | 1 |
| NL13 | 0,79 | 43 | 0 | 57 | 100 | 100 | 62 | NAO;EA;SCA;SOI | 4 | 1 |
| NL21 | 0,75 | 25 | 25 | 75 | 75 | 86 | 60 | NAO | 1 | 6 |
| NL34 | 0,75 | 17 | 33 | 83 | 67 | 71 | 80 | NAO | 1 | 1 |
| PT18 | 0,70 | 60 | 0 | 40 | 100 | 100 | 67 | EA;SCA;EAWR | 3 | 25 |
| RO12 | 0,79 | 43 | 0 | 57 | 100 | 100 | 25 | EAWR;SOI | 2 | 20 |
| RO21 | 0,86 | 29 | 0 | 71 | 100 | 100 | 33 | EAWR;SOI;EA;SCA;NAO | 5 | 20 |
| RO41 | 0,83 | 33 | 0 | 67 | 100 | 100 | 50 | EAWR | 1 | 24 |
| UKD1 | 0,88 | 25 | 0 | 75 | 100 | 100 | 89 | SOI | 1 | 39 |
| UKD3 | 0,88 | 25 | 0 | 75 | 100 | 100 | 89 | SOI | 1 | 39 |
| UKD4 | 0,88 | 25 | 0 | 75 | 100 | 100 | 89 | SOI | 1 | 39 |
| UKD6 | 0,88 | 25 | 0 | 75 | 100 | 100 | 89 | SOI | 1 | 39 |
| UKD7 | 0,88 | 25 | 0 | 75 | 100 | 100 | 89 | SOI | 1 | 39 |
| UKE1 | 0,88 | 25 | 0 | 75 | 100 | 100 | 89 | SOI | 1 | 39 |
| UKE2 | 0,88 | 25 | 0 | 75 | 100 | 100 | 89 | SOI | 1 | 39 |
| UKE3 | 0,88 | 25 | 0 | 75 | 100 | 100 | 89 | SOI | 1 | 39 |
| UKE4 | 0,88 | 25 | 0 | 75 | 100 | 100 | 89 | SOI | 1 | 39 |
| UKF1 | 0,88 | 25 | 0 | 75 | 100 | 100 | 89 | SOI | 1 | 39 |
| UKF2 | 0,88 | 25 | 0 | 75 | 100 | 100 | 89 | SOI | 1 | 39 |
| UKF3 | 0,88 | 25 | 0 | 75 | 100 | 100 | 89 | SOI | 1 | 39 |
| UKG1 | 0,88 | 25 | 0 | 75 | 100 | 100 | 89 | SOI | 1 | 39 |
| UKG2 | 0,88 | 25 | 0 | 75 | 100 | 100 | 89 | SOI | 1 | 39 |
| UKG3 | 0,88 | 25 | 0 | 75 | 100 | 100 | 89 | SOI | 1 | 39 |
| UKH1 | 0,88 | 25 | 0 | 75 | 100 | 100 | 89 | SOI | 1 | 39 |
| UKH2 | 0,88 | 25 | 0 | 75 | 100 | 100 | 89 | SOI | 1 | 39 |
| UKH3 | 0,88 | 25 | 0 | 75 | 100 | 100 | 89 | SOI | 1 | 39 |
| UKJ1 | 0,88 | 25 | 0 | 75 | 100 | 100 | 89 | SOI | 1 | 39 |
| UKJ3 | 0,88 | 25 | 0 | 75 | 100 | 100 | 89 | SOI | 1 | 39 |
| UKK1 | 0,88 | 25 | 0 | 75 | 100 | 100 | 89 | SOI | 1 | 39 |
| UKK2 | 0,88 | 25 | 0 | 75 | 100 | 100 | 89 | SOI | 1 | 39 |
| UKK3 | 0,88 | 25 | 0 | 75 | 100 | 100 | 89 | SOI | 1 | 39 |
| UKK4 | 0,88 | 25 | 0 | 75 | 100 | 100 | 89 | SOI | 1 | 39 |
| UKL1 | 0,88 | 25 | 0 | 75 | 100 | 100 | 89 | SOI | 1 | 39 |
| UKL2 | 0,88 | 25 | 0 | 75 | 100 | 100 | 89 | SOI | 1 | 39 |
| UKN0 | 0,88 | 25 | 0 | 75 | 100 | 100 | 89 | SOI | 1 | 39 |

Table S1.5. Standard classification statistics for best performing FFT at lead time 2.

| **NUTS2 ID** | **AUC** | **False Alarm (%)** | **Miss (%)** | **Correct Rejection (%)** | **Hit Rate (%)** | **Negative Predictive Value (%)** | **Positive Predictive Value (%)** | **Cue(s)** | **Number of Predictors** | **Number of Missing Values at the NUTS2 Level** |
| --- | --- | --- | --- | --- | --- | --- | --- | --- | --- | --- |
| AT12 | 0,80 | 0 | 40 | 100 | 60 | 78 | 100 | NAO;EA;SCA | 3 | 0 |
| AT31 | 0,76 | 20 | 29 | 80 | 71 | 67 | 83 | EA;NAO | 2 | 0 |
| BE21 | 0,75 | 0 | 50 | 100 | 50 | 67 | 100 | SOI;SCA;EA;NAO | 4 | 0 |
| BE22 | 0,79 | 0 | 43 | 100 | 57 | 62 | 100 | EA;EAWR | 2 | 0 |
| BE23 | 0,92 | 0 | 17 | 100 | 83 | 86 | 100 | EAWR;SOI;SCA;EA;NAO | 5 | 0 |
| BE25 | 0,75 | 50 | 0 | 50 | 100 | 100 | 67 | SCA;NAO;EA;EAWR | 4 | 0 |
| BE33 | 0,76 | 20 | 29 | 80 | 71 | 67 | 83 | EA;EAWR | 2 | 0 |
| DE13 | 0,75 | 17 | 33 | 83 | 67 | 71 | 80 | EA;SOI;EAWR;NAO;SCA | 5 | 31 |
| DE50 | 0,78 | 11 | 33 | 89 | 67 | 89 | 67 | SOI | 1 | 18 |
| DE60 | 0,80 | 0 | 40 | 100 | 60 | 78 | 100 | SOI | 1 | 17 |
| DE72 | 0,75 | 50 | 0 | 50 | 100 | 100 | 80 | SCA;NAO;EAWR;EA;SOI | 5 | 33 |
| DE80 | 0,93 | 0 | 14 | 100 | 86 | 83 | 100 | EA;SOI;EAWR;SCA | 4 | 24 |
| DEF0 | 0,83 | 20 | 14 | 80 | 86 | 80 | 86 | EAWR;SOI;SCA;NAO | 4 | 9 |
| DEG0 | 0,70 | 60 | 0 | 40 | 100 | 100 | 70 | EA;SOI;SCA | 3 | 24 |
| DK02 | 0,75 | 25 | 25 | 75 | 75 | 86 | 60 | SCA;EA;SOI;EAWR;NAO | 5 | 31 |
| EL13 | 0,83 | 0 | 33 | 100 | 67 | 75 | 100 | EAWR | 1 | 16 |
| EL24 | 0,83 | 0 | 33 | 100 | 67 | 75 | 100 | EAWR | 1 | 16 |
| ES22 | 0,75 | 25 | 25 | 75 | 75 | 86 | 60 | SOI;SCA;EA;EAWR;NAO | 5 | 3 |
| ES42 | 0,81 | 25 | 12 | 75 | 88 | 75 | 88 | SCA | 1 | 3 |
| ES43 | 0,72 | 0 | 56 | 100 | 44 | 38 | 100 | EA;SOI;SCA | 3 | 3 |
| ES61 | 0,80 | 40 | 0 | 60 | 100 | 100 | 78 | SCA;SOI;NAO | 3 | 3 |
| ES62 | 0,78 | 33 | 11 | 67 | 89 | 67 | 89 | SCA | 1 | 4 |
| FI20 | 0,75 | 33 | 17 | 67 | 83 | 67 | 83 | EA;NAO;SCA;EAWR | 4 | 20 |
| FR25 | 0,75 | 17 | 33 | 83 | 67 | 71 | 80 | EAWR;SCA;EA;SOI;NAO | 5 | 2 |
| FR26 | 0,70 | 0 | 60 | 100 | 40 | 70 | 100 | EA;SOI | 2 | 2 |
| FR43 | 0,71 | 57 | 0 | 43 | 100 | 100 | 56 | SCA;EA;EAWR | 3 | 2 |
| FR51 | 0,75 | 17 | 33 | 83 | 67 | 71 | 80 | SCA;EA;SOI;EAWR;NAO | 5 | 2 |
| FR92 | 0,75 | 50 | 0 | 50 | 100 | 100 | 78 | EAWR;SOI | 2 | 5 |
| FR94 | 0,93 | 0 | 14 | 100 | 86 | 80 | 100 | SCA;SOI | 2 | 5 |
| HU10 | 0,80 | 0 | 40 | 100 | 60 | 60 | 100 | SCA;SOI;EAWR;NAO | 4 | 20 |
| HU21 | 0,80 | 40 | 0 | 60 | 100 | 100 | 60 | EA;SOI;EAWR | 3 | 20 |
| HU23 | 0,75 | 50 | 0 | 50 | 100 | 100 | 67 | EA;SCA | 2 | 20 |
| HU33 | 0,75 | 50 | 0 | 50 | 100 | 100 | 86 | EA;NAO;SOI;SCA | 4 | 20 |
| ITF5 | 0,76 | 29 | 20 | 71 | 80 | 83 | 67 | SOI;EAWR | 2 | 6 |
| ITF6 | 0,70 | 50 | 10 | 50 | 90 | 50 | 90 | EAWR;SOI;EA;NAO | 4 | 6 |
| LV00 | 1,00 | 0 | 0 | 100 | 100 | 100 | 100 | EA;EAWR;SOI | 3 | 22 |
| NL22 | 0,94 | 0 | 12 | 100 | 88 | 80 | 100 | EAWR;SOI | 2 | 6 |
| NL41 | 0,75 | 50 | 0 | 50 | 100 | 100 | 91 | SCA;EA;NAO | 3 | 1 |
| PL42 | 1,00 | 0 | 0 | 100 | 100 | 100 | 100 | SOI;EAWR;EA;SCA;NAO | 5 | 20 |
| PL61 | 0,75 | 0 | 50 | 100 | 50 | 67 | 100 | EA;EAWR | 2 | 20 |
| PL63 | 0,73 | 33 | 20 | 67 | 80 | 67 | 80 | SOI | 1 | 20 |
| RO12 | 0,79 | 43 | 0 | 57 | 100 | 100 | 25 | EAWR | 1 | 20 |
| RO21 | 0,79 | 43 | 0 | 57 | 100 | 100 | 25 | EAWR | 1 | 20 |
| RO22 | 0,75 | 0 | 50 | 100 | 50 | 86 | 100 | EAWR;EA;SOI;SCA;NAO | 5 | 22 |
| RO31 | 0,90 | 20 | 0 | 80 | 100 | 100 | 75 | EAWR | 1 | 21 |
| RO32 | 0,83 | 33 | 0 | 67 | 100 | 100 | 50 | EA;EAWR | 2 | 25 |
| RO41 | 0,92 | 17 | 0 | 83 | 100 | 100 | 67 | EAWR | 1 | 24 |
| RO42 | 0,90 | 20 | 0 | 80 | 100 | 100 | 75 | EAWR | 1 | 20 |
| SK02 | 0,73 | 33 | 20 | 67 | 80 | 67 | 80 | EA;SOI;SCA;EAWR | 4 | 20 |

Table S1.6. Standard classification statistics for best performing FFT at lead time 1.

| **NUTS2 ID** | **AUC** | **False Alarm (%)** | **Miss (%)** | **Correct Rejection (%)** | **Hit Rate (%)** | **Negative Predictive Value (%)** | **Positive Predictive Value (%)** | **Cue(s)** | **Number of Predictors** | **Number of Missing Values at the NUTS2 Level** |
| --- | --- | --- | --- | --- | --- | --- | --- | --- | --- | --- |
| AT31 | 0,71 | 0 | 57 | 100 | 43 | 56 | 100 | EA;EAWR;SOI;SCA;NAO | 5 | 0 |
| BE10 | 0,70 | 50 | 10 | 50 | 90 | 50 | 90 | EAWR;EA;NAO;SCA;SOI | 5 | 3 |
| BE21 | 0,83 | 0 | 33 | 100 | 67 | 75 | 100 | NAO;EA;SOI | 3 | 0 |
| BE23 | 0,83 | 0 | 33 | 100 | 67 | 75 | 100 | EA;SOI | 2 | 0 |
| BE25 | 0,92 | 17 | 0 | 83 | 100 | 100 | 86 | NAO;SOI;EA;SCA;EAWR | 5 | 0 |
| DE13 | 0,75 | 50 | 0 | 50 | 100 | 100 | 67 | EA;NAO;SCA | 3 | 31 |
| DE21 | 0,75 | 50 | 0 | 50 | 100 | 100 | 80 | EA;EAWR;NAO;SCA | 4 | 31 |
| DE50 | 0,72 | 22 | 33 | 78 | 67 | 88 | 50 | SOI | 1 | 18 |
| DE80 | 0,76 | 20 | 29 | 80 | 71 | 67 | 83 | NAO;EA | 2 | 24 |
| DE93 | 0,75 | 50 | 0 | 50 | 100 | 100 | 80 | SCA;EA;EAWR;NAO | 4 | 32 |
| DEA3 | 0,75 | 50 | 0 | 50 | 100 | 100 | 67 | EA;NAO;SCA | 3 | 31 |
| DEA4 | 0,75 | 50 | 0 | 50 | 100 | 100 | 80 | EA;SCA;EAWR;NAO;SOI | 5 | 31 |
| ES22 | 0,81 | 38 | 0 | 62 | 100 | 100 | 57 | SCA;EA;EAWR;SOI;NAO | 5 | 3 |
| ES61 | 0,79 | 0 | 43 | 100 | 57 | 62 | 100 | EA;EAWR;NAO;SCA;SOI | 5 | 3 |
| FI1B | 0,86 | 0 | 29 | 100 | 71 | 50 | 100 | EA;EAWR;NAO | 3 | 39 |
| FI1C | 0,86 | 0 | 29 | 100 | 71 | 50 | 100 | EA;EAWR;NAO | 3 | 39 |
| FI1D | 0,86 | 0 | 29 | 100 | 71 | 50 | 100 | EA;EAWR;NAO | 3 | 39 |
| FR43 | 0,90 | 0 | 20 | 100 | 80 | 88 | 100 | NAO;EA;SCA;SOI;EAWR | 5 | 2 |
| IE01 | 0,88 | 0 | 25 | 100 | 75 | 67 | 100 | SCA;SOI;EAWR;EA;NAO | 5 | 38 |
| IE02 | 0,88 | 0 | 25 | 100 | 75 | 67 | 100 | SCA;SOI;EAWR;EA;NAO | 5 | 38 |
| NL22 | 0,75 | 50 | 0 | 50 | 100 | 100 | 80 | EA;EAWR;SOI;NAO | 4 | 6 |
| PL22 | 0,75 | 50 | 0 | 50 | 100 | 100 | 67 | NAO;SCA;SOI | 3 | 20 |
| PL34 | 0,92 | 0 | 17 | 100 | 83 | 67 | 100 | EA;SCA;SOI;EAWR | 4 | 20 |
| PL62 | 0,70 | 60 | 0 | 40 | 100 | 100 | 50 | NAO | 1 | 20 |
| RO21 | 0,86 | 29 | 0 | 71 | 100 | 100 | 33 | EA;SCA;SOI;EAWR;NAO | 5 | 20 |
| RO22 | 1,00 | 0 | 0 | 100 | 100 | 100 | 100 | EA;SCA;EAWR;SOI;NAO | 5 | 22 |
| RO42 | 0,83 | 0 | 33 | 100 | 67 | 83 | 100 | SOI;EA;EAWR;SCA;NAO | 5 | 20 |

**S2 Spatial distribution of the sugar beet production predictors**

**
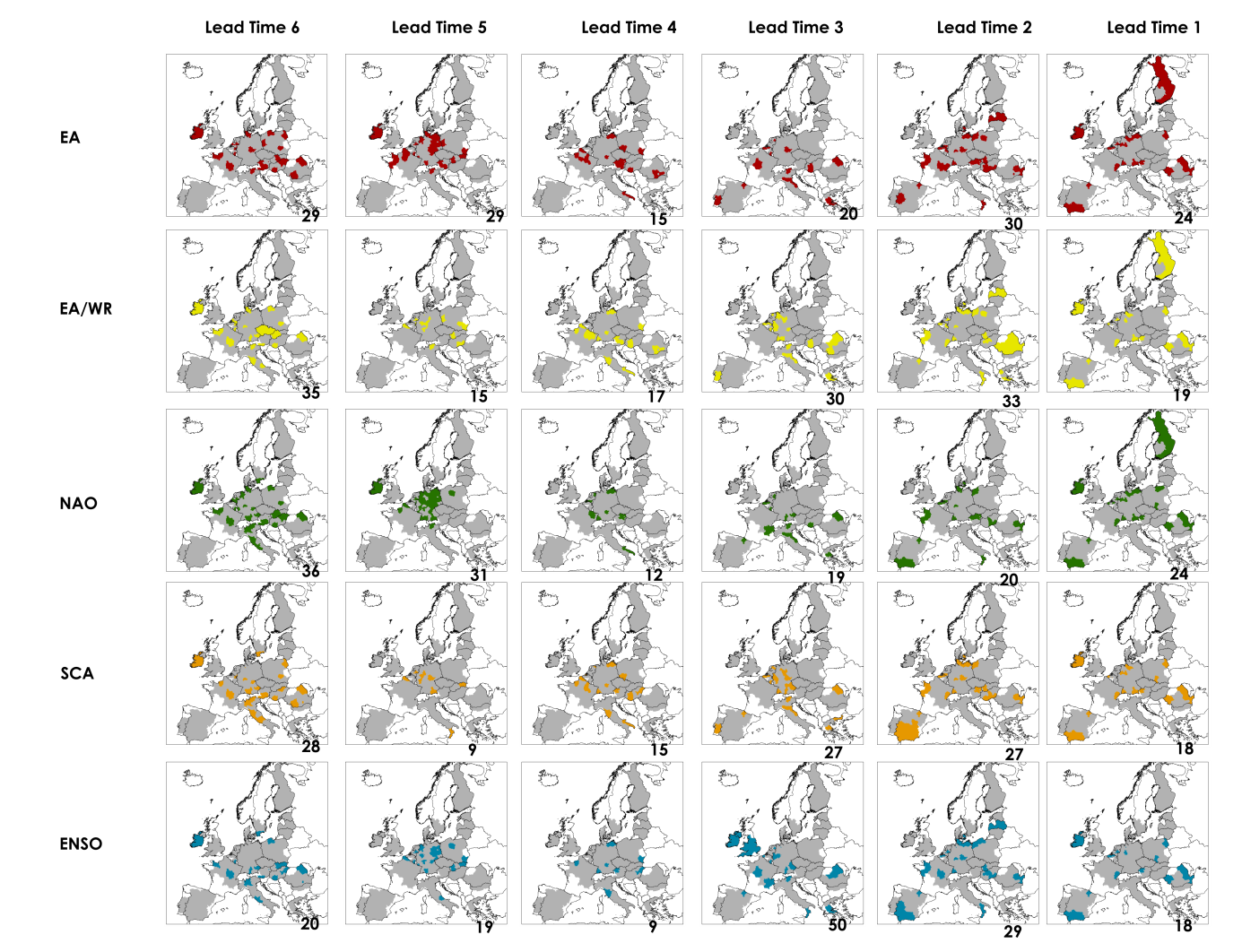
**

**Figure S2.** Spatial distribution of the indices of climate variability that were used as predictors by the FFT model for regions with significant AUC>0.7 at six lead times. Regions without predictive skill (AUC<0.7) are shown in grey, and the number at the bottom of each map the amount of times that each index was used as predictors.

**S3 Spatial distribution of the sugar beet production**

**
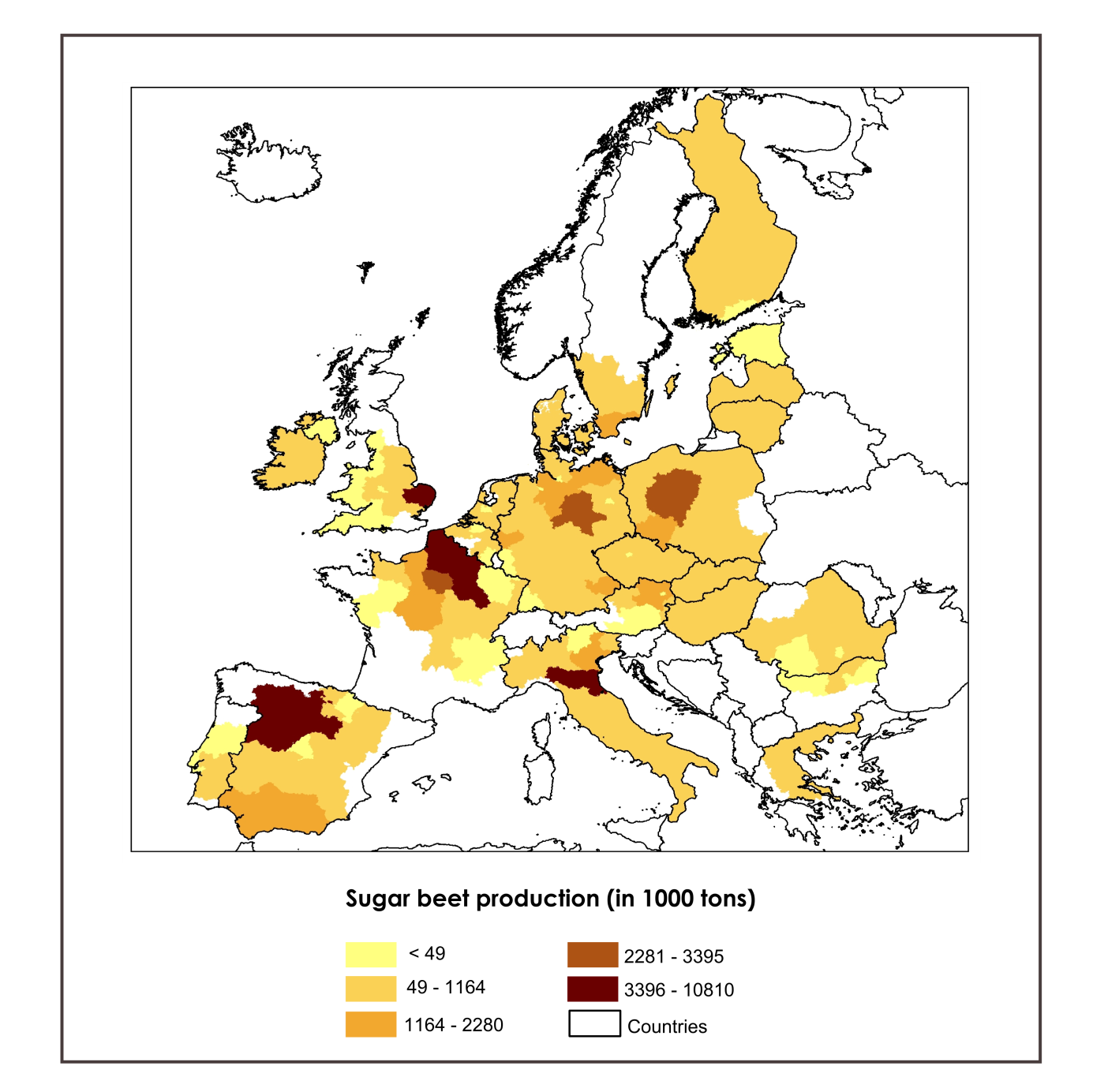
**

**Figure S3:** Distribution of the mean sugar beet production (in 1000 tons) per NUTS2 region in the investigated areas.

**S4 Spatial distribution of number of predictors**

**
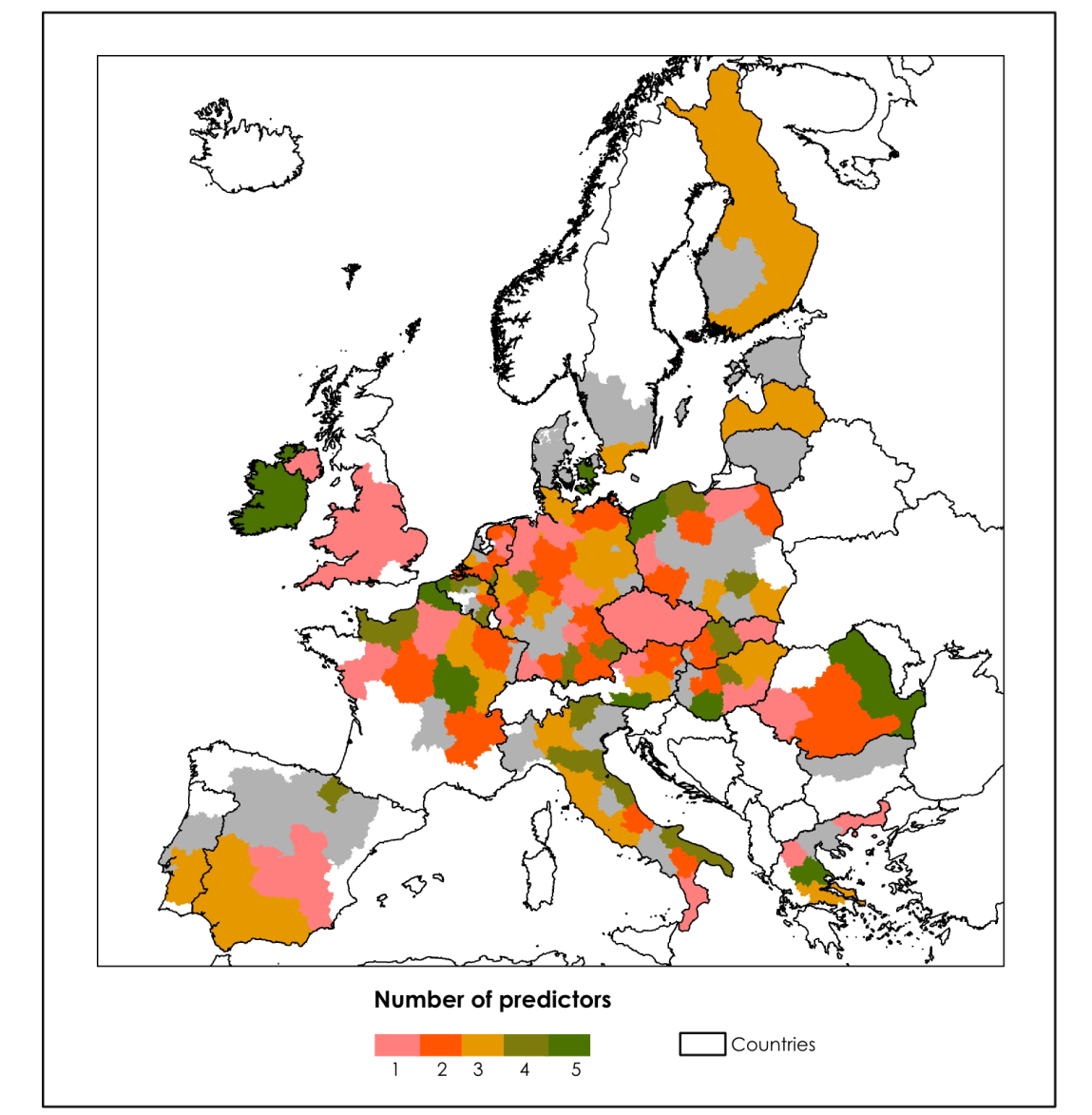
**

**Figure S4.** Spatial distribution of number of indices of climate variability that were used as predictors by the FFT model for regions with significant AUC>0.7 at six lead times. NUTS2 regions in grey represent the areas investigated. The maps were overlaid in descending order from longest to shortest lead time.

**S5 Estimate of the sugar beet harvest area**

**
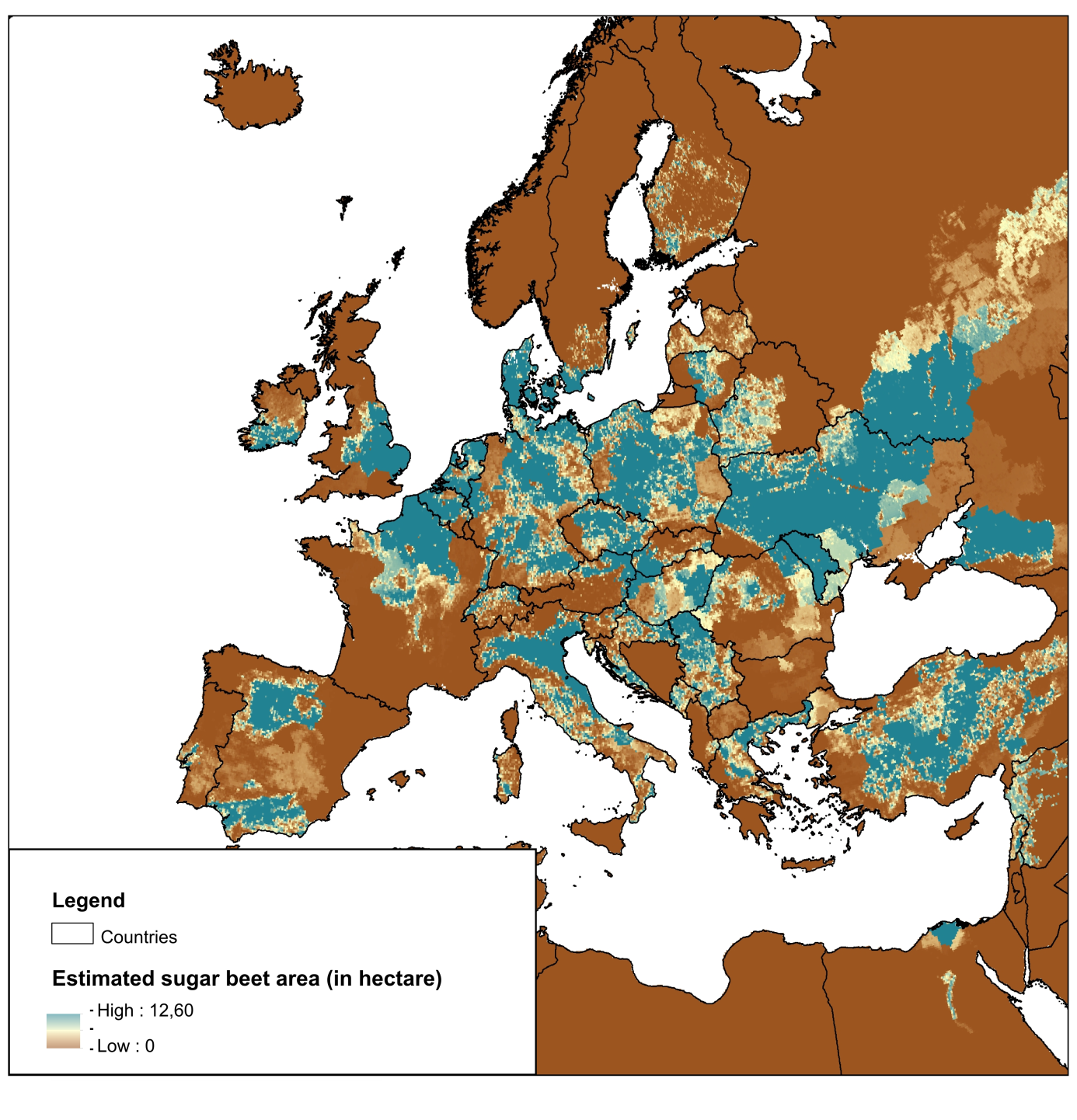
**

**Figure S5.** Estimated sugar beet area (in hectare) per grid cell (0.08° x 0.08°) obtained from the MIRCA 2000 project. For further description of dataset refer to previous studies ^1,2^. Dataset is available online at http://www.uni-frankfurt.de/45218023/MIRCA?legacy_request=1

**S6 Study case regions**

**
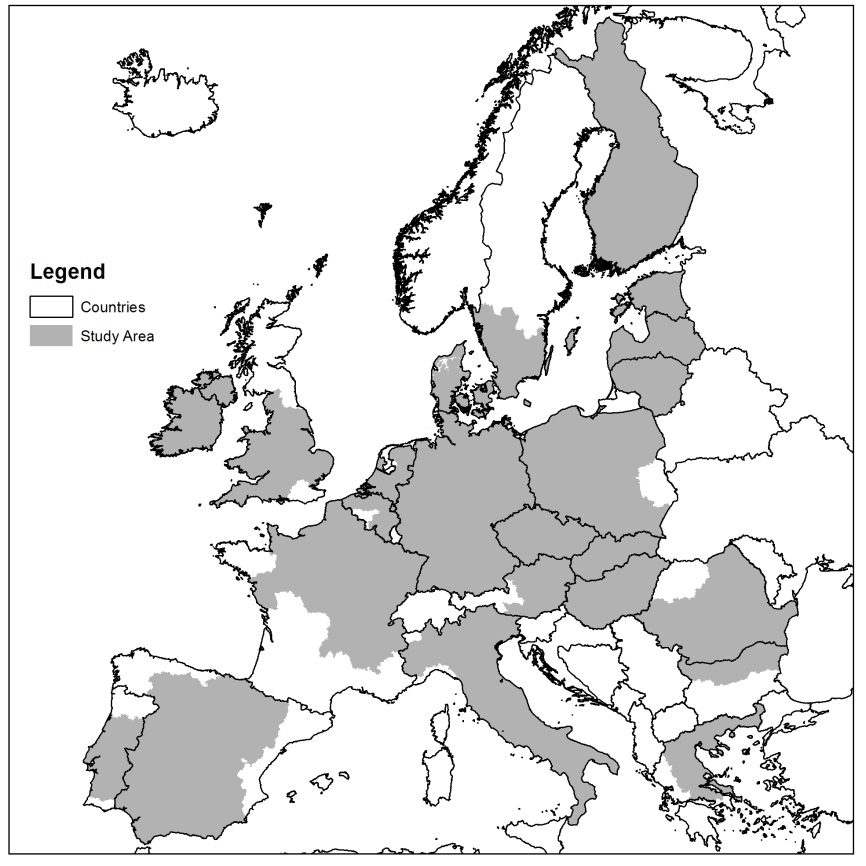
**

**Figure S6.** NUTS2 regions investigated in this study.

**S7 Summary of statistics**

Table S7. Summary of statistics presenting mean, standard deviation and length of sugar beet production dataset for 207 NUTS2 regions investigated in this study from 1975-2013.

| **NUTS2** | **Mean Value Sugar Beet Production (in 1000 tons)** | **Standard Deviation of Sugar Beet Production (in 1000 tons)** | **Length of data** | **NUTS2** | **Mean Value Sugar Beet Production (in 1000 tons)** | **Standard Deviation of Sugar Beet Production (in 1000 tons)** | **Length of data** |
| --- | --- | --- | --- | --- | --- | --- | --- |
| AT11 | 276,28 | 276,28 | 39 | FR91 | 600,65 | 600,65 | 37 |
| AT12 | 2047,30 | 2047,3 | 39 | FR92 | 182,16 | 182,16 | 37 |
| AT13 | 22,47 | 22,47 | 39 | FR93 | 4,85 | 4,85 | 37 |
| AT21 | 0,98 | 0,98 | 39 | FR94 | 1621,94 | 1621,94 | 37 |
| AT22 | 14,26 | 14,26 | 39 | HU10 | 98,99 | 98,99 | 27 |
| AT31 | 383,26 | 383,26 | 39 | HU21 | 274,40 | 274,4 | 27 |
| BE10 | 2,77 | 2,77 | 39 | HU22 | 600,86 | 600,86 | 27 |
| BE21 | 36,89 | 36,89 | 39 | HU23 | 315,51 | 315,51 | 27 |
| BE22 | 394,58 | 394,58 | 39 | HU31 | 151,64 | 151,64 | 27 |
| BE23 | 338,40 | 338,4 | 39 | HU32 | 922,16 | 922,16 | 27 |
| BE24 | 507,99 | 507,99 | 39 | HU33 | 489,58 | 489,58 | 27 |
| BE25 | 795,36 | 795,36 | 39 | IE01 | 91,71 | 91,71 | 39 |
| BE33 | 756,37 | 756,37 | 39 | IE02 | 1079,85 | 1079,85 | 39 |
| BE34 | 8,46 | 8,46 | 39 | ITC1 | 349,30 | 349,3 | 39 |
| BE35 | 670,75 | 670,75 | 39 | ITC4 | 1027,49 | 1027,49 | 39 |
| BG31 | 31,33 | 31,33 | 27 | ITF1 | 253,15 | 253,15 | 39 |
| BG32 | 78,70 | 78,7 | 27 | ITF2 | 112,30 | 112,3 | 39 |
| BG33 | 21,90 | 21,9 | 27 | ITF3 | 61,68 | 61,68 | 39 |
| CZ01 | 23,99 | 23,99 | 27 | ITF4 | 643,52 | 643,52 | 39 |
| CZ02 | 1020,14 | 1020,14 | 27 | ITF5 | 114,44 | 114,44 | 39 |
| CZ03 | 237,45 | 237,45 | 27 | ITF6 | 110,80 | 110,8 | 39 |
| CZ04 | 251,88 | 251,88 | 27 | ITH1 | 1,62 | 1,62 | 39 |
| CZ05 | 773,31 | 773,31 | 27 | ITH2 | 7,08 | 7,08 | 39 |
| CZ06 | 795,80 | 795,8 | 27 | ITH3 | 1817,47 | 1817,47 | 39 |
| CZ07 | 344,02 | 344,02 | 27 | ITH4 | 200,11 | 200,11 | 39 |
| CZ08 | 179,23 | 179,23 | 27 | ITH5 | 4073,35 | 4073,35 | 39 |
| DE11 | 755,29 | 755,29 | 39 | ITI1 | 271,24 | 271,24 | 39 |
| DE12 | 277,68 | 277,68 | 39 | ITI2 | 142,78 | 142,78 | 39 |
| DE13 | 28,01 | 28,01 | 39 | ITI3 | 1139,66 | 1139,66 | 39 |
| DE14 | 63,45 | 63,45 | 39 | ITI4 | 220,10 | 220,1 | 39 |
| DE21 | 531,53 | 531,53 | 39 | LT00 | 840,75 | 840,75 | 27 |
| DE22 | 1197,99 | 1197,99 | 39 | LV00 | 315,42 | 315,42 | 27 |
| DE23 | 330,27 | 330,27 | 39 | NL11 | 798,15 | 798,15 | 39 |
| DE24 | 93,61 | 93,61 | 39 | NL12 | 246,91 | 246,91 | 39 |
| DE25 | 355,81 | 355,81 | 39 | NL13 | 751,28 | 751,28 | 39 |
| DE26 | 1083,94 | 1083,94 | 39 | NL21 | 125,71 | 125,71 | 39 |
| DE27 | 451,22 | 451,22 | 39 | NL22 | 243,42 | 243,42 | 39 |
| DE30 | 0,64 | 0,64 | 39 | NL31 | 15,29 | 15,29 | 39 |
| DE40 | 702,26 | 702,26 | 39 | NL32 | 450,96 | 450,96 | 39 |
| DE50 | 0,70 | 0,7 | 39 | NL33 | 486,50 | 486,5 | 39 |
| DE60 | 2,20 | 2,2 | 39 | NL34 | 939,97 | 939,97 | 39 |
| DE71 | 553,28 | 553,28 | 39 | NL41 | 765,20 | 765,2 | 39 |
| DE72 | 79,93 | 79,93 | 39 | NL42 | 646,99 | 646,99 | 39 |
| DE73 | 315,70 | 315,7 | 39 | PL11 | 519,81 | 519,81 | 27 |
| DE80 | 1274,25 | 1274,25 | 39 | PL12 | 1022,96 | 1022,96 | 27 |
| DE91 | 2360,38 | 2360,38 | 39 | PL21 | 92,80 | 92,8 | 27 |
| DE92 | 1921,12 | 1921,12 | 39 | PL22 | 159,68 | 159,68 | 27 |
| DE93 | 1373,65 | 1373,65 | 39 | PL32 | 298,47 | 298,47 | 27 |
| DE94 | 154,78 | 154,78 | 39 | PL33 | 421,40 | 421,4 | 27 |
| DEA1 | 1022,11 | 1022,11 | 39 | PL34 | 214,15 | 214,15 | 27 |
| DEA2 | 2067,79 | 2067,79 | 39 | PL41 | 2478,34 | 2478,34 | 27 |
| DEA3 | 85,05 | 85,05 | 39 | PL42 | 646,76 | 646,76 | 27 |
| DEA4 | 361,25 | 361,25 | 39 | PL43 | 129,82 | 129,82 | 27 |
| DEA5 | 155,91 | 155,91 | 39 | PL51 | 1311,76 | 1311,76 | 27 |
| DEB1 | 138,06 | 138,06 | 39 | PL52 | 1001,29 | 1001,29 | 27 |
| DEB2 | 31,45 | 31,45 | 39 | PL61 | 2293,47 | 2293,47 | 27 |
| DEB3 | 1012,11 | 1012,11 | 39 | PL62 | 240,94 | 240,94 | 27 |
| DEC0 | 5,85 | 5,85 | 39 | PL63 | 599,05 | 599,05 | 27 |
| DED2 | 302,06 | 302,06 | 39 | PT16 | 9,91 | 9,91 | 37 |
| DED4 | 169,39 | 169,39 | 39 | PT17 | 14,74 | 14,74 | 37 |
| DED5 | 404,66 | 404,66 | 39 | PT18 | 111,06 | 111,06 | 37 |
| DEE0 | 2558,20 | 2558,2 | 39 | PT20 | 16,50 | 16,5 | 39 |
| DEF0 | 753,51 | 753,51 | 39 | RO12 | 422,81 | 422,81 | 27 |
| DEG0 | 623,89 | 623,89 | 39 | RO21 | 673,22 | 673,22 | 27 |
| DK01 | 91,57 | 91,57 | 39 | RO22 | 177,39 | 177,39 | 27 |
| DK02 | 872,27 | 872,27 | 39 | RO31 | 117,94 | 117,94 | 27 |
| DK03 | 855,21 | 855,21 | 39 | RO32 | 1,11 | 1,11 | 27 |
| DK04 | 814,13 | 814,13 | 39 | RO41 | 37,41 | 37,41 | 27 |
| DK05 | 386,18 | 386,18 | 39 | RO42 | 220,47 | 220,47 | 27 |
| EE00 | 1,54 | 1,54 | 26 | SE21 | 206,24 | 206,24 | 30 |
| EL11 | 717,67 | 717,67 | 39 | SE22 | 2102,53 | 2102,53 | 30 |
| EL12 | 767,65 | 767,65 | 39 | SE23 | 53,02 | 53,02 | 30 |
| EL13 | 182,59 | 182,59 | 39 | SK01 | 53,24 | 53,24 | 27 |
| EL14 | 427,69 | 427,69 | 39 | SK02 | 1127,16 | 1127,16 | 27 |
| EL24 | 93,00 | 93 | 39 | SK03 | 53,35 | 53,35 | 27 |
| ES21 | 144,25 | 144,25 | 39 | SK04 | 71,69 | 71,69 | 27 |
| ES22 | 26,39 | 26,39 | 39 | UKD1 | 9,82 | 9,82 | 39 |
| ES23 | 156,48 | 156,48 | 39 | UKD3 | 3,57 | 3,57 | 39 |
| ES24 | 63,63 | 63,63 | 39 | UKD4 | 12,41 | 12,41 | 39 |
| ES30 | 3,04 | 3,04 | 39 | UKD6 | 6,46 | 6,46 | 39 |
| ES41 | 3749,21 | 3749,21 | 39 | UKD7 | 1,26 | 1,26 | 39 |
| ES42 | 555,70 | 555,7 | 39 | UKE1 | 276,69 | 276,69 | 39 |
| ES43 | 99,26 | 99,26 | 39 | UKE2 | 391,99 | 391,99 | 39 |
| ES61 | 1973,30 | 1973,3 | 39 | UKE3 | 66,02 | 66,02 | 39 |
| ES62 | 62,04 | 62,04 | 39 | UKE4 | 70,45 | 70,45 | 39 |
| FI19 | 290,77 | 290,77 | 30 | UKF1 | 325,78 | 325,78 | 39 |
| FI1B | 0,90 | 0,9 | 30 | UKF2 | 533,68 | 533,68 | 39 |
| FI1C | 289,65 | 289,65 | 30 | UKF3 | 648,75 | 648,75 | 39 |
| FI1D | 290,80 | 290,8 | 30 | UKG1 | 380,14 | 380,14 | 39 |
| FI20 | 17,36 | 17,36 | 30 | UKG2 | 243,24 | 243,24 | 39 |
| FR10 | 3001,08 | 3001,08 | 39 | UKG3 | 24,30 | 24,3 | 39 |
| FR21 | 6641,02 | 6641,02 | 39 | UKH1 | 3906,58 | 3906,58 | 39 |
| FR22 | 10810,33 | 10810,33 | 39 | UKH2 | 364,88 | 364,88 | 39 |
| FR23 | 1717,46 | 1717,46 | 39 | UKH3 | 663,21 | 663,21 | 39 |
| FR24 | 1953,61 | 1953,61 | 39 | UKJ1 | 300,71 | 300,71 | 39 |
| FR25 | 546,24 | 546,24 | 39 | UKJ3 | 6,89 | 6,89 | 39 |
| FR26 | 442,75 | 442,75 | 39 | UKK1 | 42,98 | 42,98 | 39 |
| FR30 | 4378,33 | 4378,33 | 39 | UKK2 | 8,63 | 8,63 | 39 |
| FR41 | 20,35 | 20,35 | 39 | UKK3 | 3,69 | 3,69 | 39 |
| FR42 | 360,29 | 360,29 | 39 | UKK4 | 6,87 | 6,87 | 39 |
| FR43 | 60,59 | 60,59 | 39 | UKL1 | 2,27 | 2,27 | 39 |
| FR51 | 41,51 | 41,51 | 39 | UKL2 | 11,21 | 11,21 | 39 |
| FR71 | 14,00 | 14 | 39 | UKN0 | 0,62 | 0,62 | 39 |
| FR72 | 251,10 | 251,1 | 39 |  |  |  |  |

**S8 Train-test split method**

We cross-validated and pruned the FFTs models using the train-test split method, which we describe below in five steps. Pruning is a technique in machine learning that reduces the size of decision trees by removing predictors that provide little decision power to the model.

1. Set the maximum number of predictors levels equal to 5, and train the FFTs models in 70% of all data.
2. Test the model in the other 30% remaining;
3. Calculate the balanced accuracy of the tested model;
4. Repeat step 1-3 four times adopting a maximum predictors levels equal to 4, 3, 2 and 1, subsequently;
5. Select the model and the pruning parameter (predictors levels) that maximizes the balanced accuracy of the FFTs models.

**S9 The AUC index**


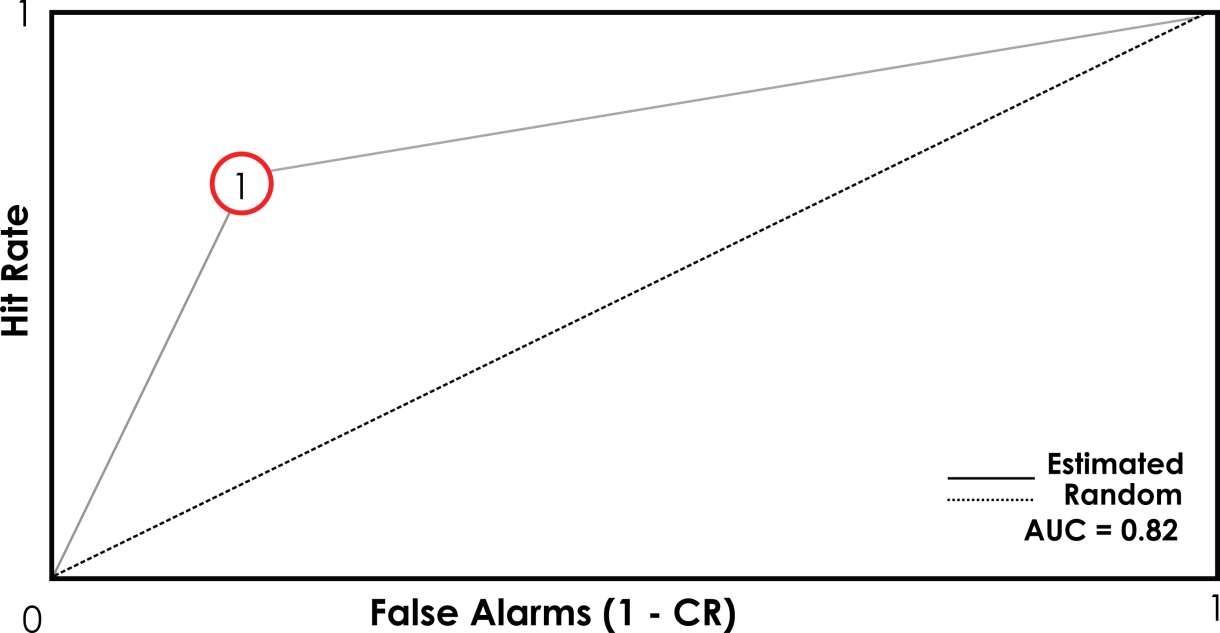


**Figure S9.** Representation of the AUC index calculated using the trapezoidal rule. The AUC index measures how well the FFT model can distinguish between two classes (low/high). A random classification migrates towards the diagonal dashed line.

**S10 Bootstrapping and assessing the significance of the AUC index**

We tested the significance of the AUC index at the NUTS2 level using a bootstrapping method, which we describe in 5 steps. The bootstrapping method is applied after we obtain the FFT model and the pruning parameter (described in supplementary S8) that maximizes the balanced accuracy. We apply bootstrapping to test whether the AUC index of a random series is higher than AUC index obtained from the original series. Lastly, we assess the spatial significance of the results by applying a Field Significance test using the binomial distribution^3^. This test addresses the null hypothesis that the number of significant regions is different to the number of regions that one would expect by chance.

1. First, we bootstrap with replacement to obtain 1,000 samples of binary high and low sugar beet production events of length referent to the number of years of each NUTS2 region. For example, for the NUTS2 region AT11 and each lead time, we randomly selected 39 values 1,000 times as there are 39 years of sugar beet production available;
2. Second, we split the random series into training (70%) and testing (30%) data, similarly as applied in the original series. For example, for NUTS2 region AT11 the 1,000 training data each have a length of 27 values, while the 1,000 testing data have each a length of 12 values.
3. Third, for each NUTS2 region, lead time and testing data, we fitted our best performing FFT model. Next, we calculate for each NUTS2 region and lead time, 1,000 AUC indices of the tested models.
4. Fourth, for each NUTS2 region and lead time, we ranked the 1,000 AUC indices, and obtained the 90% percentile value (α = 10 %) from the distribution of 1,000 AUC indices. Lastly, we compared this value to the one obtained in the original series.
5. Fifth, we assessed the large-scale significance of the results by applying a Field Significance test using the binomial distribution. The results that were found not to be highly significant (P < 0.001) are indicated in Figure 1 with an asterisk. In other words, regions that were found to be significant only due bootstrap test are indicated with an asterisk in Figure 1.

**References**

1. Sacks, W. J., Deryng, D. & Foley, J. A. Crop planting dates : an analysis of global patterns. *Glob. Ecol. Biogeogr.* **19,** 607–620 (2010).

2. Portmann, F. T., Siebert, S. & Döll, P. MIRCA2000-Global monthly irrigated and rainfed crop areas around the year 2000: A new high-resolution data set for agricultural and hydrological modeling. *Global Biogeochem. Cycles* (2010). doi:10.1029/2008GB003435

3. Livezey, R. E. & Chen, W. Y. Statistical Field Significance and its Determination by Monte Carlo Techniques. *Monthly Weather Review* **111,** 46–59 (1983).
